# Supplementary material for: Perceptions, experiences and barriers to lifestyle modifications in first-generation Middle Eastern immigrants to Sweden: a qualitative study
Source: BMJ Open. 2019 Oct 19;9(10):e028076. doi: 10.1136/bmjopen-2018-028076 (PMC6803114; doi:10.1136/bmjopen-2018-028076)
Supplement: Supplementary data [file bmjopen-2018-028076supp001.pdf]

Group sessions the MEDIM Intervention study.

The intervention is modified from "The Image toolkit for prevention of type 2 diabetes in Europe: "Take action to prevent diabetes" http://www.image-project.eu/pdf/final_version_of_toolkit-perfect.pdf.

Questionnaires:

- Stage of change [2] or motivation level to enroll in an intervention study, to loose weight and increase their physical activity
- QALY EQ-5D [3]
- Questions about lifestyle habits (diet & exercise, psychological wellbeing)
- Background questions: socioeconomy, current medication and previous diseases, heredity regarding diabetes and cardiovascular diseases, acculturation
- Self-determination theory [4]
- SMART goals that form the basis for the Action Plan Work Sheet (will be developed)
- Physical activity diary
- Food diary

Additional comments:
Smartphone reminders as well as a "Support Line" via telephone.

Group meetings are recorded on tape to document what is said at meetings during discussion.

Session 1. To increase the awareness and knowledge of how lifestyle habits predicts risk of diabetes and cardiovascular disease. Identify health and unhealthy lifestyle habits

A. Identify present lifestyle habits:

1. **Let them fill out questionnaires regarding their present food and physical activity (PA) habits. Discuss their answers**

1. **To increase the awareness and knowledge of how lifestyle habits (food, PA, smoking) predicts risk of diabetes and cardiovascular disease.**
   a. Inform and discuss healthy level of PA and food intake.
   b. Discuss their answers from the questionnaires and ask the participants to identify healthy and unhealthy lifestyle habits regarding food and PA habits.

Session 2. ”To stimulate motivation”:

1. **To stimulate motivation**
    a. Let the participants describe lifestyle habits that they need to change. If they can’t identify them, help them.
2. **Motivation**
    a. Identify and discuss barriers to lifestyle change
    b. Discuss strategies how to handle barriers
    c. Discuss the importance of social support
    d. Discuss pros and cons to lifestyle change (“motivation cross” used in Primary health care)
    e. Identify how important it is to them to change their lifestyle habits and let them fill out the form “Stage of change”
    f. Ask if
        i. They want to continue participating in the intervention
        ii. They want to think about it (participate in the same group again)
        iii. They don’t want to continue (drop)
    g. Give them homework, to take a walk 15 minutes per day.
    h. Provide them with an Action plan work sheet and ask them to set a short term goal, when to do the activity, for how long and how often and what social support they can get. For example take a walk 15 minutes a day when the children are back from school so they can take care of their little 1 year old brother.

Session 3. ”To get started/Taking Action”

Goals:

1. **To create an action plan**
2. **To handle and reduce the risk of relapse**
3. **Homework**

1. **To create an action plan**
    a. Ask the participants to identify lifestyle habits that they want to change
    b. Ask them to fill in the Stage of change form
    c. If they are ready for change ask them what changes they want to do.
    d. Introduce the SMART goal theory and practice to set short and long term goals (physical activity 150 min/week and 5% weight reduction from start). Make them create a ladder of short term goals that will ultimately lead to the big goal. The plan should also include social support they need to achieve and what coping strategies they will use if necessary.

e. Ask participants to write down their own action plan. (This action plan may be revised as they reach their short term goals. A diploma as they reach their short term goals, other ideas of rewards?)

2. To handle and reduce the risk of relapse

a. Discuss the behavioral change wheel, and explain that set backs are a part of the process and an opportunity to learn

b. Discuss and provide tools how to handle and deal with set backs

c. Discuss and provide tools how to handle negative thoughts and stress

d. Identify barriers to change and facilitators for lifestyle change. Identify things that are or are not helping them reaching their goals.

3. Homework

a. Practice action planning, identify negative thoughts and then counter with positive thoughts.

Session four: Cooking session

Session 5 to 7. ”Maintaining lifestyle change”

Goals

1. **Review the progress of lifestyle change**
2. **Review the behavioral change process**
3. **Repeat how to handle relapse**
4. **Rewrite the action plan and set new goals**

1. Review the progress of lifestyle change

a. Go through the homework. What changes have they made? What barriers have they encountered? How did they deal with these barriers? What facilitated to reach goals?

b. Review the SMART goals and their Action plan work sheet. Focus on goals they have achieved. Emphasize the importance of self-monitoring and ask participants to evaluate their progress using their diaries, action plans and smart goals.

2. **Review the behavioral change process and the motivation for behavioral change.** Ask them if it is important to them to implement change and why. Let them fill in a stage of change form.

3. Repeat how to handle relapse.

a. Support successes and discuss setbacks and use them as educational opportunities. Have they made use of coping strategies? If, what has worked? What has not worked? Identify the obstacles that the participants experienced. Discuss in small groups of obstacles that were most relevant to them (emotional, cost-oriented, knowledge-related). In the group, discuss how to get past these barriers. Go through the large group what has been taken up in the small groups. Emphasize the importance of self-monitoring (for example the

use of the accelerometer, etc.). Ask the participants to reflect on what has worked and what has not worked. Make sure the focus is on reaching milestones so that it is easier to build self-confidence and opportunities to achieve success. Ask participants to reflect on their expectations of what to achieve are realistic.

b. Illustrate the importance of periodically review goals and how they evolved. Which goals are they most pleased to have reached? Have there been any unexpected positive changes? Ask participants to identify ways that they can reward themselves.

c. Ask participants to identify positive and negative sources of social support that they had experienced. Did the social support work that they put into their action plan? If not, why? Ask the group to develop more ideas for how social support can act.

4. Rewrite the action plan and set new goals
