# Supplementary material for: Perceptions, experiences and barriers to lifestyle modifications in first-generation Middle Eastern immigrants to Sweden: a qualitative study
Source: BMJ Open. 2019 Oct 19;9(10):e028076. doi: 10.1136/bmjopen-2018-028076 (PMC6803114; doi:10.1136/bmjopen-2018-028076)
Supplement: Author's manuscript [file bmjopen-2018-028076.draft_revisions.pdf]

BMJ Open

BMJ Open is committed to open peer review. As part of this commitment we make the peer review history of every article we publish publicly available.

When an article is published we post the peer reviewers' comments and the authors' responses online. We also post the versions of the paper that were used during peer review. These are the versions that the peer review comments apply to.

The versions of the paper that follow are the versions that were submitted during the peer review process. They are not the versions of record or the final published versions. They should not be cited or distributed as the published version of this manuscript.

BMJ Open is an open access journal and the full, final, typeset and author-corrected version of record of the manuscript is available on our site with no access controls, subscription charges or pay-per-view fees (http://bmjopen.bmj.com).

If you have any questions on BMJ Open's open peer review process please email info.bmjopen@bmj.com

BMJ Open

Perceptions, experiences and barriers to lifestyle modifications in first-generation Middle Eastern immigrants to Sweden

Journal:	*BMJ Open*
Manuscript ID	bmjopen-2018-028076
Article Type:	Research
Date Submitted by the Author:	29-Nov-2018
Complete List of Authors:	Olaya-Contreras, Patricia; Sahlgrenska Academy, University of Gothenburg,, Institute of Health and Care Sciences<br>Balcker-Lundgren, Katarina; Center for Primary Health Care Research, Skåne University Hospital, Malmö, Sweden<br>Siddiqui , Faiza ; Lunds Universitet, Department of Clinical Sciences; Center for Primary Health Care Research, Skåne University Hospital, Malmö, Sweden<br>Bennet, Louise; Lund University, Center for Primary Health Care Research; Lund University, Malmö, Sweden
Keywords:	Type-2-Diabetes, Thematic Analysis, lifestyle modifications, obesogenic-environments, Middle-Eastern-immigrants

SCHOLARONE™
Manuscripts

Manuscript title: Perceptions, experiences and barriers to lifestyle modifications in first-generation Middle Eastern immigrants to Sweden

Running title: Lifestyle modifications in Middle Eastern immigrants to Sweden

Word count: 4250 words

Olaya-Contreras, P[1], Balcker, K[2], Siddiqui, F[2,3], Bennet, L[2,3].

[1]Institute of Health and Care Sciences, Sahlgrenska Academy, University of Gothenburg, Sweden.

[2] Center for Primary Health Care Research, Skåne University Hospital, Malmö, Sweden

[3] Department of Clinical Sciences, Lund University, Malmö, Sweden

Corresponding author

Patricia Olaya-Contreras

Institute of Health and Care Sciences, Sahlgrenska Academy, University of Gothenburg, Sweden.

Box 457, 405 30 Göteborg, Sweden

Patricia.olaya-contreras@gu.se

Phone number +46707 450383

Funding

This work was supported by grants from Lund University (ALF grants Dnr 20101641, 20101837, and 162641), Region Skåne (Dnr 226661 and 121811), the Swedish Society of Medicine (Dnr 97081 and 176831), the Crafoord Foundation (Dnr 20110719), the Swedish Research Council Linné grant to LUDC (Dnr 349-2006-237 and Dnr 349-2008-6589), Strategic Research area Exodiab (Dnr 2009-1039), Swedish Foundation for Strategic Research (Dnr IRC15-0067) and ANDIS (Dnr 825-2010-5983).

"We have read and understood BMJ policy on declaration of interests and declare that we have no competing interests."

For peer review only - http://bmjopen.bmj.com/site/about/guidelines.xhtml

AUTHOR STATEMENT

POC: Wrote the manuscript, analysed the data and translated the material coming from the group discussions from Swedish to English.

KB: Participated in conducting the group sessions. Participated in data acquisition and data assessment. Contributed with interpretation of the data. Participated in writing the manuscript.

FS: Participated in the design of the study and in conducting the group sessions. Participated in assessing data and interpretation of the data interpretation. Contributed to writing the manuscript.

LB: Designed and conducted the original study, contributed data acquisition, data assessment and interpretation of the data. Participated in writing the manuscript.

For peer review only

Acknowledgements

We are grateful to health coach Muna Mohammed, translator ImanYousif, study Nurses Saranda Muhaxheri, Josefin Goode Khan, and Asma Saleh, administrator Sonja Ruhnke and Ida Hagman. We are also grateful to Hälsans Hus, Rosengård, Abena, Viktväktarna, Arbetarnas Bildningsförbund (ABF), and Oatly. We also thank Josephine T. V. Greenbrook for her help with the English language in the final draft of the manuscript.

Data sharing Statement: Data can be made available upon request to the corresponding author.

For peer review only - http://bmjopen.bmj.com/site/about/guidelines.xhtml

Abstract

Word count: 272

The aim of the present study was to explore perceptions, experiences, and barriers concerning lifestyle modifications in Middle-Eastern immigrants to Sweden.

Design: A qualitative thematic analysis was conducted on data collected from gender-specific focus group interviews which took place during a culturally adapted randomized controlled intervention study addressing motivation to lifestyle change, self-empowerment, behavioural modifications, and sociocultural barriers to lifestyle modification. Seven focus groups were held, with an interval of 1 – 4 weeks between January and May of 2015; each session lasted approximately 1.5 hours.

Setting: The study was conducted in Malmö, Sweden.

Participants Nine women and 14 men who had attended at least one focus group session were engaged in the sessions.

Results: Participants expressed awareness surrounding the content of healthy lifestyle practices, however, perceived numerous sociocultural barriers to lifestyle modifications, connected to irregular meals, food and drinking preferences, food preparation, family expectations, and cultural norms. Overeating, with large portion sizes, was the main barrier to improving eating-habits. Participants explained overeating as a consequence of sociocultural norms and expectations, and as a result of poor mental wellbeing. The women perceived lack of family support as a barrier for lifestyle modification, whereas facilitators for successful lifestyle modifications were connected to family involvement and support throughout the intervention.

Conclusion: Identification of barriers and facilitators for lifestyles modifications in ethnic minority populations at increased risk for T2D is crucial for both health care professionals and society to successfully support these populations in preventative efforts. The present study highlighted that

For peer review only

facilitators for lifestyles modifications were connected to profound family support. Further research should focus on exploring community networks and caregivers' interactions when coaching groups exposed to "obesogenic environments".

Keywords: Type-2-Diabetes, Qualitative Thematic Analysis, lifestyle modifications, obesogenic-environments, Middle-Eastern-immigrants

For peer review only

For peer review only - http://bmjopen.bmj.com/site/about/guidelines.xhtml

Article Summary

Strengths and limitations of the study

- A strength of this study is the use of focus group discussions, which encourages interaction between respondents to explore and clarify individual and shared perspectives on lifestyle modifications and views on the intervention.
- To address language barriers, a native Arabic speaker was present in all sessions who performed back- and forward translations when transcriptions were done.
- Perspectives on sociocultural barriers to lifestyle modification of Iraqi men immigrants in Sweden, or those of younger Iraqi immigrant women and men, are not represented in this study.
- Cultural perceptions and norms may limit the women´ physical activities, as expressed throughout the discussions.

For peer review only

For peer review only - http://bmjopen.bmj.com/site/about/guidelines.xhtml

Background

Political instability in the Middle East during recent decades has forced millions of people to flee their homelands. A large proportion of these migrants reach Europe and in Sweden today first generation immigrants from the Middle East constitute over 400000 individuals, and the largest non-European population in the country, with the largest groups born in Iraq or Syria. [1]

Non-western immigrants to Europe are often referred to live in socioeconomically deprived areas thus leading to higher morbidity and mortality rates. [2–3] Poor socioeconomic conditions contribute to higher diabetes incidence. For instance, in Sweden, refugees assigned to live in neighbourhoods of high deprivation developed type 2 diabetes (T2D) to a higher extent compared to those assigned to a neighbourhood of low deprivation. [4] The prevalence of T2D among Iraqi immigrants is almost twice as high as compared to that of the native Swedish population. [5] Despite more doctor visits and the fact that they receive pharmacological treatment at an earlier stage, non-western immigrants have worse metabolic control, and develop diabetes complications at an earlier stage, compared to native Swedes. [6]

Structural and sociocultural norms influence lifestyle practices and are correlated with a high risk for T2D. [7–8] In different geographical contexts, immigrants and refugees have a higher risk than the native population to develop T2D, which has been associated with eating calorie dense foods and physical inactivity. [9–11] Lifestyle interventions conducted in westernized populations prevents T2D to a higher extent than pharmacological therapy with the hypoglycaemic agent 'metformin'. [12] However, in ethnic minority populations, lifestyle interventions are not successful and individuals at risk do not reach target unless interventions are culturally adapted. [11] Few qualitative studies have focused on studying the effect of cultural and social factors on lifestyle modifications (LSM) in

For peer review only - http://bmjopen.bmj.com/site/about/guidelines.xhtml

ethnic minority populations. To understand barriers and facilitators to LSM non-westernized immigrants are exposed to, this study attempted to investigate the influence of cultural- and social factors on lifestyle habits from the perspectives of Middle Eastern immigrants to Sweden.

The aim of the present study was to explore perceptions, experiences and barriers to LSM in Iraqi immigrants who are overweight and at high risk of T2D. Increased understanding of barriers and facilitators to LSM are crucial in the design of relevant preventive support addressing high-risk to T2D among non-European immigrant populations.

Methods

The present study used a qualitative research design in the collection and analysis of data based on a culturally adapted intervention, the MEDIM cohort study. [13]

Patient and Public Involvement

The development of the research question. They all origin from the Middle East i.e. another culture where lifestyle is governed by traditions and social norms to a higher extent than in the western culture. Several prior studies have posited that in non-western populations there are sociocultural barriers to lifestyle change, but the knowledge of what these barriers look like are not well known or understood.

Recruitment of participants: Participants identified at risk for type 2 diabetes (n=1398), i.e., with overweight, BMI >28 kg/m2, in the population based MEDIM study 2010-2012, were eligible to participate in this study. [13] In 2015, out of these 1398 individuals, 636 completely filled the requirements to be included in the intervention. [13] Of them, 96 consent to participate in this study. [13.] Half of the participants, 27 women and 23 men, were randomly assigned to the intervention group

For peer review only - http://bmjopen.bmj.com/site/about/guidelines.xhtml

(n=50). Participants had been residing in Sweden for several years in the city of Malmö, where Iraqis constitute the largest group of immigrants. The majority of the participants held lower education levels, less than the secondary school (86%), and almost half were unemployed (52 %). Most women were married, with children between 2 to 6 per family. All spoke Arabic, and were unable to communicate in Swedish. Out of 50 participants, 19 women and 14 men, who had attended at least one focus group session, were included in the present study (Table 1). Half of the male participants participated in only one session; however, 14% attended almost all sessions (Figure 1). The main reason for non-participation among them was not being given leave from work. None of the participants were not known to the investigators prior to their involvement in the study. Patients were not involved in the recruitment nor design of this study.

Procedure

The intervention group was offered to participate in a total of seven gender-specific focus group interviews, which were conducted in their native language. The sessions addressed awareness, motivation, taking action, relapse, review and feedback, setting goals, and self-empowerment (Table 2). The interview guides for focus group sessions included structured and open-ended questions to explore a specific set of topics; First, by asking broader questions surrounding difficulties in reaching the set goal, and subsequently asking focal questions specific to each session (See Table 2). The interview guides were developed based on the evidence-based guidelines for prevention of T2D, [13] and were pilot-tested many times prior to the intervention. Participants individually answered the interviewer's questions, and were encouraged to interact with each other to further explore views on the intervention. An interdisciplinary team (a specialist nurse in diabetes and a health coach) conducted the focus groups with assistance from an Arabic/Swedish interpreter. Each session lasted approximately 1.5 hours, with intervals of 1–4 weeks between sessions, over a period of four months. All sessions were audio recorded. The interview recordings were

For peer review only - http://bmjopen.bmj.com/site/about/guidelines.xhtml

subsequently translated by an official translator from Arabic to Swedish, and transcribed verbatim. Thereafter, transcripts were checked against the recordings for accuracy. All authors were responsible for reviewing the translated data, transcripts, and notes for data analyses.

Dissemination of findings: The findings will be disseminated by publications, interviews, lectures and media.

Data analysis

In analysing the data gathered from the focus group interviews, a deductive thematic analysis was conducted. The approach is an independent and reliable qualitative approach to analysis which provides a purely qualitative, detailed, and nuanced account of the data [14–15]. A modified version of Braun and Clarke's [14] thematic analysis model was implemented. The data was organized to extract emergent patterns in the semantic content. All of the authors read the transcribed data multiple times. A thematic codebook was developed to facilitate identification of main themes, allowing for the multiple coverage of possible topics. The first author made codes by marking important features in the transcribed text, and sorted data relevant to each code. The authors met to discuss the codes, in order to identify themes potentially converging or diverging, and further, to explore possible additional meanings in the content of participants' narratives [14]. Major themes were defined as those responses/opinions voiced by more than half of the participants. NVivo 10 software was used in the coding process [16].

Ethical considerations

All participants provided voluntary written informed consent prior to their participation in the present study. They were informed about the possibility to withdraw from the study without explanation and without consequences. The Ethical Review Board of Lund University, Sweden

For peer review only - http://bmjopen.bmj.com/site/about/guidelines.xhtml

approved the study (approval no. 2011/88). All data collected was anonymised, and stored on a protected server only accessible by selected members of the research team.

3. Results

The findings are condensed into three Themes: (A) Motivation to lifestyle modifications ─ Awareness of diet habits and physical activities related to health ─; (B) Perceptions, experiences and barriers towards healthier lifestyles; and (C) Facilitators for maintaining lifestyle modifications.

3.1 Motivation to lifestyle modifications: Awareness of diet habits and physical activities related to health

According to the participants, healthy lifestyles were understood as being physically active, i.e. doing sport/exercise, going to the gym regularly, eating vegetables and fruits, fibre rich- and balanced diet. Some testimonies illustrate what healthy factors meant to them:

> *To avoid sweets such as cookies, instead to drink water; To eat vegetables, as much as possible; To be physically active at least half an hour every day; To walk often; To reduce weight; You should sleep at least 7 hours; You should eat cooked food; One should not eat fried food; You should drink a lot of water and eat fish; You should eat regularly.*

They were aware of unhealthy lifestyles:

For peer review only - http://bmjopen.bmj.com/site/about/guidelines.xhtml

To consume too much red meat; to be obese or overweight; to drink soft drinks; to eat candies; to get stressed

Physical inactivity was associated with illness, causing diseases among them that already existed among their relatives, as illustrated below:

The reason why I have been physically active is to lose weight; I do not want a stroke or diabetes; many in my family suffer from diabetes, so, I do not want to suffer from it.

3.2 Perceptions, experiences and barriers towards healthier lifestyles

3.2.1 The challenge with regular meals

The participants described that they had difficulties having regular meals such as breakfast, lunch and dinner, often skipping the main meals, and commonly replacing meals with sweets. Some of the women explained that the reason was because the children ate at school, and when mothers felt hungry they provided themselves with some snacks or fast food:

I do not usually eat so much lunch, I eat some chocolate when I get tired in the afternoon; I eat when I feel hungry, I do not think so much about time to eat; I eat many meals often. At home, sometimes I go walking and eat, I have no specific time to eat.

Among them, it was also common to have late dinner, i.e. at night, sometimes overeat or, on the contrary, they ate almost nothing.

3.2.2 Food preparation, taste and portion size

For peer review only - http://bmjopen.bmj.com/site/about/guidelines.xhtml

Fried or sweet tasting foods were preferred and recognized as "*food with good smell and taste*", which they described as highly appreciated. Some of the testimonies exemplified this issue:

> *You do not feel good if you do not eat fried foods; I believe that I eat more fried products after walking because I need it*

Some participants did not like the taste of the water; instead they drank sodas and/or sweet juices. Other disliked the taste of foods boiled in water and cooked instead of fried. A common statement was they consumed neither integral products with fibre rich flour nor low-calorie products. The ways to prepare foods or the kind of ingredients used were perceived by the women as the most difficult to change:

> *I found it very difficult to change our diet, from the food that we like to eat, but I thought, I would be able to consume less sweet products*

Participants liked to eat large portions. They had a habit of serving large portions, eating many fried snacks, sweet beverages (sodas and tea with sugar), and candies. Some women believed that they were overweight because of how much they had eaten in each meal, and how much food they had eaten between meals, rather than because of how the food was prepared:

> *I realize that the problem, for me, it is the amount of food and the portion size.*

<u>*3.2.3 Family traditions and expectations*</u>

A common statement was that the children decided what and where to eat, which explained some of the difficulties to change eating-habits. The mothers were eating fast food as children did because of "*the need to follow family decisions*" and, in addition, the women explained their overeating because "*the boys need to eat enormous portions after exercising in the evening*". As a result of this, they prepared a lot of foods, and therefore, they were serving themselves big portions

For peer review only - http://bmjopen.bmj.com/site/about/guidelines.xhtml

afterwards. Nevertheless, some of the women mentioned that after eating in excess they become sad or upset with themselves. Some families drank soda everyday (at least four cans each per day) but the mothers were concerned with this; they expressed that it needed to be stopped.

It was described that as part of family traditions ─ women must follow the relative's manner to eat when celebrating and meeting relatives. This implies to eat copiously as the rest of the family does when attending parties or dinners.

> *Yes, when I meet relatives, they invite me [to eat] all the time, and I cannot say no…; this is considered impolite not to eat the food when offered; It is hard to say no to good food that you get served; My family thinks I'm too small, but I'm overweight! They offer me different dishes and meals the whole time*

The women expressed interest in exercising but they felt alone in this effort. They did not feel support from their family members for exercising, and they described they did not have free time for outdoors physical activity, or for going to the gym. Women commonly described that their time was dedicated to household chores and time with the children. It was clearly stated that, as a mother, you must play a role and prioritize it:

> *I like to exercise but it's hard to find time when I have children; In my family, my children and my husband are not engaged in exercise and thus, I will be alone in my effort to exercise; I live in Sweden, I do not work, I take care of the household, children, shopping and cooking and have no time for sports; It´s difficult to get time for exercise, but I try to be physically active as often as it is possible.*

3.2.4 Climate and other external factors influencing motivation level

The participants expressed that they struggled to adapt to the cold and dark climate, which had a negative influence on outdoors PA, as one of the participants illustrated:

For peer review only - http://bmjopen.bmj.com/site/about/guidelines.xhtml

I get depressed when I eat less...; The weather is boring and the darkness too and I do not have so many social contacts; When I cook and the kids do not eat that much, I begin eating instead of exercising

It was stated that after some years without exercise, it was difficult to establish a routine and feel motivated to exercise regularly, and this feeling was worse when suffering from pain. One woman explained:

I have lived in Sweden for 8 years, I have four children, and I have not exercised in these years.

One male participant preferred to make other activities at home that did not require physical activity:

Even though I'm on sick-leave, I do not have time to exercise.

3.2.5. The paradox: raised awareness but still no lifestyle modifications

Despite the raised consciousness concerning the health benefits from changing dietary habits and increasing level of physical activity, –especially the women – described that they continued eating sweets and pastries mainly when they were alone, and copiously, large portions, when sharing with family and relatives. They found it difficult to lose weight, which they experienced, was related to social/family pressure, as mentioned above.

In fact, some of the women disliked go to the gym, they considered the gym as a “stuffy place”; further, they stated it was difficult to be alone there.

We would like to go to places only for women where we can exercise and do sports. These facilities need to be located near where we live; otherwise, it is not possible for us to go there.

For peer review only

3.3. Facilitators for maintaining lifestyle modifications

3.3.1 Changing lifestyle habits together

In some of the families, the husbands were involved and supported the modifications implemented by their wife. In other families, the mothers began controlling their children's intake of sugar, and others described that the sessions influenced the whole family, i.e. preparing some dishes in a healthier way:

> *The whole family has changed dietary habits; My husband eats only hard rye bread now and no white bread; We eat a lot of vegetables, not like before; We bought whole grain rice and started using it instead; My husband has begun to check calories and ingredients in the food he buys, and now he is aware of the food we should not consume and those we should*

3.3.2 Involvement of family members and socializing through healthier habits

Some participants in the intervention group described that they took regular walks together with family members, some started going to the gym:

> *I have started to walk a lot and be more physically active; I have started with water aerobics three times a week and working out twice a week; I also walk; I'm doing gymnastics, training 1 hour each day, 3 times a week and really, I feel better inside and outside; I had tried the treadmill and have trained with the machines in the gym. It is necessary to be strong to be able to lose weight and to get a healthier life*

Two of the women tried to involve their family members in domestic tasks, facilitating their possibility of having some extra time for PA:

For peer review only - http://bmjopen.bmj.com/site/about/guidelines.xhtml

My daughter and I walk together but my steps are more than hers are. I'm walking fast, I usually walk from 11 o´clock to 17; My son is practicing every day with me in the gym, so he helps me; I have lost 5-6 kilos, I think

During the intervention, some participants lost weight after increasing their everyday physical activity, despite some health problems, which is illustrated here:

Before, I weighed 77 kilos, and now I weigh 70 kilos; My goal was weight loss and although I have pain in my knee, I lost 10 kg; I have walked, and I need to continue walking or exercising to lose weight; I have focused on physical training and if I cannot walk, I swim or exercise at home

Discussion

This study is one of the first qualitative studies investigating self-perceived experiences, barriers and facilitators to LSM in Middle Eastern immigrant populations to Europe, at high risk for T2D. Our participants expressed awareness of the content of healthy lifestyles and identified sociocultural barriers to LSM connected to; lack of gender-specific facilities, lack of time, food preparation and preferences, lack of regular meals, cold and dark climate, as well as poor mental health. Family expectations and structural problems were mentioned by females as a strong contributor to maintaining unhealthy lifestyle practices. A key finding was that facilitators for successful LSM were connected to family involvement and social-environmental support.

The participants perceived that over time it was difficult to establish new dietary habits and become more physically active, which is consistent with studies showing that individuals living in so-called "obesogenic environments", [17] challenges in achieving modifications are often hindered by

For peer review only

For peer review only - http://bmjopen.bmj.com/site/about/guidelines.xhtml

contextual and families influences. [17–19] Availability for exercise centres, arenas and other physical environments are important contributors to lifestyles changes. [7, 11, 17, 20–24] In Malmö, there are several gyms for women and in this study, "Hälsans Hus" i.e. gym facilities only for women located in the nearby neighbourhood was one of the physical activity centres women were referred to. Nevertheless, the perceived lack of nearby gender specific gym facilities was a common reason among women to continue being physically inactive. Lack of time was an additional reason for not performing regular physical activity in this study. A previous Swedish study showed that women wanted non- strenuous 'suitable and pleasant' exercise, i.e., cycling, swimming, and gymnastics. Women, particularly from Iraq and Turkey, expressed that it is important that the exercise is 'proper' i.e. not to exercise in front of the men, and exercise that was initiated and arranged by the school or the workplace but not by themselves. [22] Hence, close collaboration with their communities/family is mandatory to fully understand and adjust physical activity for different needs among female immigrants with T2D, as confirmed by previous research. [9, 11, 21, 22–24]

The perceptions that food, which isn't stir fried, or unsweetened drinks do not taste good is a challenge to conquer, especially since eating patterns are commonly guided by family and children's expectations as referred to in the interviews. We tried to address this by having discussions addressing knowledge gaps regarding how lifestyle influences the risk of developing T2D. However, the participants perceived as difficult to modify dietary preferences, especially when sharing with family and friends, in accordance with prior research. [17, 19–21]

In the intervention, 10 000 steps per day was set as a daily goal, but it was perceived for most participants, as unreachable. Beginning with lower step-goals can enhance adherence to the intervention in sedentary groups. [9] Thus, in this study, a reason for not complied with the intervention could be due to the perception that the goals were too difficult to reach.

For peer review only - http://bmjopen.bmj.com/site/about/guidelines.xhtml

Participants experienced difficulties to modify their lifestyles. The women experienced that in order to cope with the dark cold climate and depressed mood they overate and preferred snacks instead of regular meals, which is in line with prior studies conducted among immigrant women at risk for T2D. [2, 6, 18–19, 21] Iraqi immigrants in Sweden and Pakistani immigrants in Norway show high prevalence of poor mental health and a strong connection between anxiety and depression with physical inactivity. [25–26] Even though a cold dark climate can contribute to poor mental health as stated by our participants, neighbourhood deprivation linked to economic insecurity has also a major impact on mental health among immigrants. [27] Several studies confirm that immigrants coming from war-related circumstances face sociocultural and family changes in their new lifestyle context and have a higher risk of developing unhealthy practices. [2–3, 6–7, 21] Women commonly described feeling alone in their efforts to undergo LSM. They feel that family expectations/cultural codes such as taking care of the children and managing the household had a negative influence on their ability to be physically active, which is consistent with what has been reported in previous findings. [2, 7–11, 15, 18–23, 25]

Different theoretical models link socioeconomic- and cultural factors with poor health. For instance, in the 'Transitional Model', Schlossberg concludes that sets of factors related to contextual- and individual factors as well as environmental support seem to affect how one copes with transition and adaption to a new lifestyle. [28–29] Moreover, the World Health Organization postulates that 'social determinants', i.e., social- and community networks, working and living conditions, socioeconomic-cultural and environmental conditions, impact health on different levels. [30] Furthermore, the term 'Social capital' has been suggested to be associated with health through several different pathways, including psychosocial mechanisms, norms regarding health related behaviours and access to health care, factors that at the population level are important for the maintenance of health and reduced mortality rates. [31] Middle Eastern immigrants to Sweden in

For peer review only - http://bmjopen.bmj.com/site/about/guidelines.xhtml

general and Middle Eastern women, in particular, are exposed to all these different levels of social determinants, which is reflected by the fact that >60% of first-generation Iraqi immigrants in Malmö report low social capital (assessed as low social participation, low social anchorage, low instrumental- and low emotional support) [32], as well as high rates of morbidity in non-communicable diseases and poor mental health. Thus, low participation in this study could be a reflection of poor social capital, with low societal trust. According to a recent study, 70% of Iraqi born immigrants in Malmö do not read or write in Swedish, despite living in Sweden for more than two decades, and prefer traditional foods i.e. cook and eat foods associated with their country of origin, reflecting low levels of integration and acculturation to the new culture. [32] A strong influence of the Middle Eastern culture, on one hand contributes with health promoting factors such as strong individual family support, but on the other hand imposes cultural and social norms on each individual, as expressed by women in this study, being a strong barrier for successful LSM.

The participants who reached the goals described a profound support and involvement of their family members in this effort, i.e. helping to motivate each other to maintain healthier practices at home. These findings correspond with previous research, in which, involving family in target interventions promotes healthier eating behaviours, increased physical activity as well as wellbeing among immigrant populations. [4, 8, 11, 17–21, 27] The Transition Model highlights the importance of receiving support from both, family and environment to enhance adaptation, i.e. to cope with the intervention. Individuals move through the transition process, through a series of phases, i.e. "moving in," "moving through," and "moving out", [28–29] which may apply to our participants who need to cope, adapt, and make decisions across some cultural- and social situations to succeed with LSM.

For peer review only - http://bmjopen.bmj.com/site/about/guidelines.xhtml

Some strengths of the study are: Group discussions are a useful way to encourage respondents to explore individual and shared perspectives and views on the intervention and focus attention on possibilities to improve healthy lifestyles. A native Arabic speaker was present in all sessions, and led back- and forward translations when transcriptions were performed, which may assess accuracy of the information. A main limitation is low participation and drop out of participants. Our data may be biased by the fact that the reflections on barriers to LSM are related to middle aged rather than younger participants. Some women might fear to express themselves openly and might fear being judged if they perform outdoor physical activity. Hence, perceived barriers to lifestyle change might be underreported. Similar cultural barriers affecting participation in health interventions have been previously described among immigrants living in the US and in Sweden. [7–8, 22]

Conclusions

Identification of sociocultural barriers and facilitators for lifestyle modifications in ethnic minority populations at increased risk of T2D is crucial for both health care professionals and society in order to successfully support these populations in preventive efforts. The present study highlighted that facilitators for LSM were connected to profound family support. Tailoring interventions to include entire families, i.e., possibilities for childcare, and the involvement of the couple/other significant community members in the intervention could be successful strategies for increasing adherence to interventions/programs among Middle Eastern immigrants. To identify barriers and facilitators for LSM among this group is crucial in the understanding of the effects of an intervention, and in further acknowledging the need for continued efforts promoting healthier practices in minority populations at risk for non-communicable diseases. Further research should focus on exploring community networks and caregivers' interactions when coaching groups exposed to "obesogenic environments".

For peer review only - http://bmjopen.bmj.com/site/about/guidelines.xhtml

References

1. http://www.scb.se/sv_/Hitta-statistik/Statistik-efter-amne/Befolkning/Befolkningens-sammansattning/Befolkningsstatistik/25788/25795/Behallare-for-Press/399296/. 2018.

2. Sancho´n-Macias MaV, Bover-Bover, A., Prieto-Salceda, D, Paz-Zulueta, M., Torres B, Gastaldo D. Determinants of Subjective Social Status and Health Among Latin American Women Immigrants in Spain: A Qualitative Approach. J Immigrant Minority Health; 2016:18:436–441 DOI 10.1007/s10903-015-0197-x

3. Jervelund, S., Jervelund, S.S., Malik, S., Ahlmark, N. et al. J Immigrant Minority Health. Morbidity, Self-Perceived Health and Mortality Among non-Western Immigrants and Their Descendants in Denmark in a Life Phase Perspective. 2017;19: 448. https://doi.org/10.1007/s10903-016-0347-9

4. White JS, Hamad R, Li X, Basu S, Ohlsson H, Sundquist J, Sundquist K. Long-term effects of neighbourhood deprivation on diabetes risk: quasi-experimental evidence from a refugee dispersal policy in Sweden. Lancet Diabetes Endocrinol. 2016 Jun;4(6):517-24. doi: 10.1016/S2213-8587(16)30009-2. Epub 2016 Apr 27. PMID: 27131930)

5. Bennet L, Groop L, Lindblad U, Agardh CD, Franks PW. Ethnicity is an independent risk indicator when estimating diabetes risk with FINDRISC scores: a cross sectional study comparing immigrants from the Middle East and native swedes. Prim Care Diabetes; 2014:8(3):231–8. doi: 10.1016/j.pcd.2014.01.002. Epub 2014 Jan 25.

For peer review only - http://bmjopen.bmj.com/site/about/guidelines.xhtml

6. Rawshani A, Svensson AM, Rosengren A, Zethelius B, Eliasson B, Gudbjörnsdottir S. Impact of ethnicity on progress of glycaemic control in 131,935 newly diagnosed patients with type 2 diabetes: a nationwide observational study from the Swedish National Diabetes Register. BMJ Open. 2015 Jun 5;5(6): e007599. doi: 10.1136/bmjopen-2015-007599.

7. Njeru, JW., Formea, CM., Osman, A, Goodson, M, Hared, A, Capetillo, GP, Nigon, JA., Cha, SS., Weis, JA., Hanza, MMK., Patten, CA., Sia, IG., and Wieland, ML. Diabetes Knowledge, Attitudes and Behaviors Among Somali and Latino Immigrants. J Immigrant Minority Health; 2016: 18:1432–1440. DOI 10.1007/s10903-015-0335-5

8. Jaber LA, Pinelli NR, Brown MB, Funnell MM, Anderson R, Hammad A, Herman WH. Feasibility of group lifestyle intervention for diabetes prevention in Arab Americans. Diabetes Res Clin Pract. 2011; 91:307–315. doi: 10.1016/j.diabres.2010.11.032.

9. Musto A., Jacobs K., Nash iV, DelRossi G., and Perry A. The Effects of an Incremental Approach to 10,000 Steps/Day on Metabolic Syndrome Components in Sedentary Overweight Women. Journal of Physical Activity. 2010: Volume 7 Issue 6, November. https://doi.org/10.1123/jpah.7.6.737

10. Tiedje K, Mark L Wieland ML, Meiers SJ, Mohamed JJ, Formea CM, Ridgeway JL, Asiedu GB, Boyum G, Weis JA, Nigon JA, Patten CA and Sia GI. A focus group study of healthy eating knowledge, practices, and barriers among adult and adolescent immigrants and refugees in the

For peer review only

For peer review only - http://bmjopen.bmj.com/site/about/guidelines.xhtml

United States. Int. J Behav Nutr and Physical Activity; 2014:11:63. http://www.ijbnpa.org/content/11/1/63

11. Lirussi, F. The global challenge of type 2 diabetes and the strategies for response in ethnic minority groups. Diabetes Metab Res Rev. 2010; 26:421–432. doi: 10.1002/dmrr.1105.

12. Diabetes Prevention Program Research Group. Reduction in the incidence of type 2 diabetes with lifestyle intervention or metformin. N Engl J Med. 2002; 346:393–403. DOI: 10.1056/NEJMoa012512

13. Siddiqui, F., Kurbasic, A., Lindblad, U., Nilsson, P.M., Bennet, L. Effects of a culturally adapted lifestyle intervention on cardio-metabolic outcomes: a randomized controlled trial in Iraqi immigrants to Sweden at high risk for Type 2 diabetes. Metabolism. 2017: Volume 66, January, Pages 1-13. ISSN 0026-0495, http://dx.doi.org/10.1016/j.metabol.2016.10.001.

14. Braun, V. & Clarke, V. Using thematic analysis in psychology. Qual Res Psychol 2006;3:2, 77-101, DOI: 10.1191/1478088706qp063oa

15. Vaismoradi, M., Turunen, H. and Bondas, T. Qualitative descriptive study. Nurs Health Sci, 2013;15: 398-405. doi:10.1111/nhs.12048 https://doi.org/10.1111/nhs.12048

For peer review only

For peer review only - http://bmjopen.bmj.com/site/about/guidelines.xhtml

16. NVivo 10 [software program]. Version 10. QSR International; 2012" American Journal of Pharmaceutical Education vol. 78,1 (2014): 25.

17. Schneider S, Diehl K, Görig T, Schilling L, De Bock F, Hoffmann, K, Albrecht M, Sonntag D, and Fischer J. Contextual influences on physical activity and eating habits -options for action on the community level. 2017. BMC Public Health; 17:760 DOI 10.1186/s12889-017-4790-x

18. Schilling, L., Bock DeF, Hoffmann K, Albrecht M, Sonntag D and Fischer J. Contextual influences on physical activity and eating habits -options for action on the community level. BMC Public HealthBMC series – open 2017. https://doi.org/10.1186/s12889-017-4790-x

19. Pollard, SL., Drew A. Zachary, Katherine Wingert, Sara S. Booker, Pamela J. Surkan. Family and Community Influences on Diabetes-Related Dietary Change in a Low-Income Urban Neighborhood. The Diabetes Educator. 2014 March 31: Vol 40, Issue 4, pp. 462 – 469. https://doi-org.ezproxy.ub.gu.se/10.1177/0145721714527520

20. Everson-Hock ES, Johnson M, Jones R, Woods HB, Goyder E, Payne N, Chilcott J. Community-based dietary and physical activity interventions in low socioeconomic groups in the UK: a mixed methods systematic review. Prev Med. 2013 May;56(5):265-72. doi: 10.1016/j.ypmed.2013.02.023. Epub 2013 Feb 27.

For peer review only - http://bmjopen.bmj.com/site/about/guidelines.xhtml

21. Wieland ML, Weis JA, Palmer T, Goodson M, Loth S, Omer F, Abbenyi A, Krucker K, Edens K, Sia IG. Physical activity and nutrition among immigrant and refugee women: a community-based participatory research approach. Womens Health Issues. 2012 Mar; 22(2):e225-32. Epub 2011 Dec 11. doi: 10.1016/j.whi.2011.10.002. Epub 2011 Dec 11.

22. Södergren M1, Hylander I, Törnkvist L, Sundquist J, Sundquist K. Arranging appropriate activities immigrant women's ideas of enabling exercise. Womens Health Issues. 2008 Sep-Oct;18(5):413-22. doi: 10.1016/j.whi.2008.05.001. Epub 2008 Jul 26.

23. Wieland ML, Weis JA, Hanza MM, Meiers SJ, Patten CA, Clark MM, Sloan JA, Novotny PJ, Njeru JW, Abbenyi A, et al. Healthy immigrant families: Participatory development and baseline characteristics of a community-based physical activity and nutrition intervention. Contemp Clin Trials. 2016 Mar; 47:22-31. doi: 10.1016/j.cct.2015.12.004. Epub 2015 Dec 4.

24. Cleland CL, Mark A. Tully MA, Kee F, Margaret E. Cupples ME. The effectiveness of physical activity interventions in socio-economically disadvantaged communities: A systematic review. 2012. Preventive Medicine 54: 371–380. doi:10.1016/j.ypmed.2012.04.004

25. Siddiqui, F., Lindblad, U., and Bennet, L. Physical inactivity is strongly associated with anxiety and depression in Iraqi immigrants to Sweden: a cross-sectional study. BMC Public Health. 2014, 14:502. http://www.biomedcentral.com/1471-2458/14/502

For peer review only

26. Hjellset, VT, Bjørge B, Eriksen HR., Høstmark AT. Risk Factors for Type 2 Diabetes Among Female Pakistani Immigrants: The InvaDiab-DEPLAN Study on Pakistani Immigrant Women Living in Oslo, Norway. J Immigrant Minority Health; 2011: 13:101–110. DOI 10.1007/s10903-009-9290-3.

27. Durbin A, Moineddin R, Lin E, et al. Examining the relationship between neighborhood, deprivation and mental health service use of immigrants in Ontario, Canada: a cross-sectional study. BMJ Open 2015;5:e006690. doi:10.1136/bmjopen-2014-006690

28. Schlossberg, N. K.. A model for analyzing human adaptation to transition. The Counseling Psychologist. 1981;9(2), 2-18. http://dx.doi.org/10.1177/001100008100900202

29. Anderson, M.L., Goodman, J., & Schlossberg, N.K.. Counseling adults in transition: Linking Schlossberg's theory to practice in a diverse world (4th Ed.). 2012. New York: Springer.

30. World Health Organization. Our cities, our health, our future: acting on social determinants for health equity in urban settings. Report to the WHO Commission on Social Determinants of Health from the Knowledge Network on Urban Settings2008 (available at http://www.who.int/social_determinants/resources/knus_final_report_052008.pdf, last assessed 20181029)

31. Kawachi I, Kennedy BP, Lochner K, et al. . Social capital, income inequality, and mortality. Am J Public Health. 1997;87:1491–8.

For peer review only

For peer review only - http://bmjopen.bmj.com/site/about/guidelines.xhtml

32. Bennet Lindström. Self-rated health and social capital in Iraqi immigrants to Sweden: The MEDIM population-based study. Scand J Public Health. 2018: Mar;46(2):194-203. doi: 10.1177/1403494817730997. Epub 2017 Sep 15.

For peer review only

For peer review only - http://bmjopen.bmj.com/site/about/guidelines.xhtml

Table 1.

**Session**	**Women**	**Men**	**Total/session**
Session One: Awareness	14	9	23
Session Two: Motivation/getting started	15	7	22
Session Three: Taking Action /Maintaining the change	14	4	18
Session Four: Cooking session	9	7	16
Session Five: (Relapse – Maintaining the change)	9	3	12
Session Six: Review and feedback)	9	4	13
Session Seven: Setting goals and Self-empowerment	7	3	10

Legend: Group sessions with number of female and male participants throughout the intervention

For peer review only

Table 2.

Session one (Awareness)	The group discussion addressed how unhealthy lifestyle habits predict risk of diabetes and cardiovascular disease, as well as discussions on nutritional contents of different foods.
Session two (Motivation/Getting started)	It focused on understanding the participant's current dietary and PA habits. The *Action Plan* was introduced as a tool that helped to formulate goals for lifestyle change and to support motivation for implementing lifestyle change, i.e. To write the personal goal and describing how a participant would reach it.
Session three (Taking Action /Maintaining the change)	It was designed to get started by putting dietary change into an Action Plan, by stimulating motivation for dietary change and PA and further by discussing how to achieve the postulated goals. The session also included discussions on common cooking methods for Iraqi food and how to make dishes healthier, i.e. consisting of less fat, sugar and more fibres.
Session four (Cooking session)	In the cooking session, participants were encouraged to bring the recipe of their favourite dish. This recipe was then modified by a professional chef at the cooking session to include healthier dishes including more fibres, less fat as well as less carbohydrates in the diet. The goal of this session was to highlight how cooking method, food preparation and portion size were vital components for effective lifestyle change.
Session five (Relapse – Maintaining the change)	To follow-up the action plan, and to identify barriers to its implementation, as well as experiences regarding social support, and family engagement toward the intervention. Facilitated rewrote action plan.
Session six (Review and feedback)	Participants had documented their everyday PA in a diary and this session included discussions on the final outcomes of lifestyle change, importance of maintaining lifestyle change, motivation to set new goals and discussions on external social support/network to reach those goals. The session also included feedback from the participants.
Session seven (Setting goals and Self-empowerment)	This session highlighted the importance of regularly setting new goals and reviewing these goals. This session also focused on empowering the participants and addressed how they had handled barriers and how they had evolved and reached their goals throughout the process.

Legend: Content of the discussions at each focus group session

For peer review only - http://bmjopen.bmj.com/site/about/guidelines.xhtml

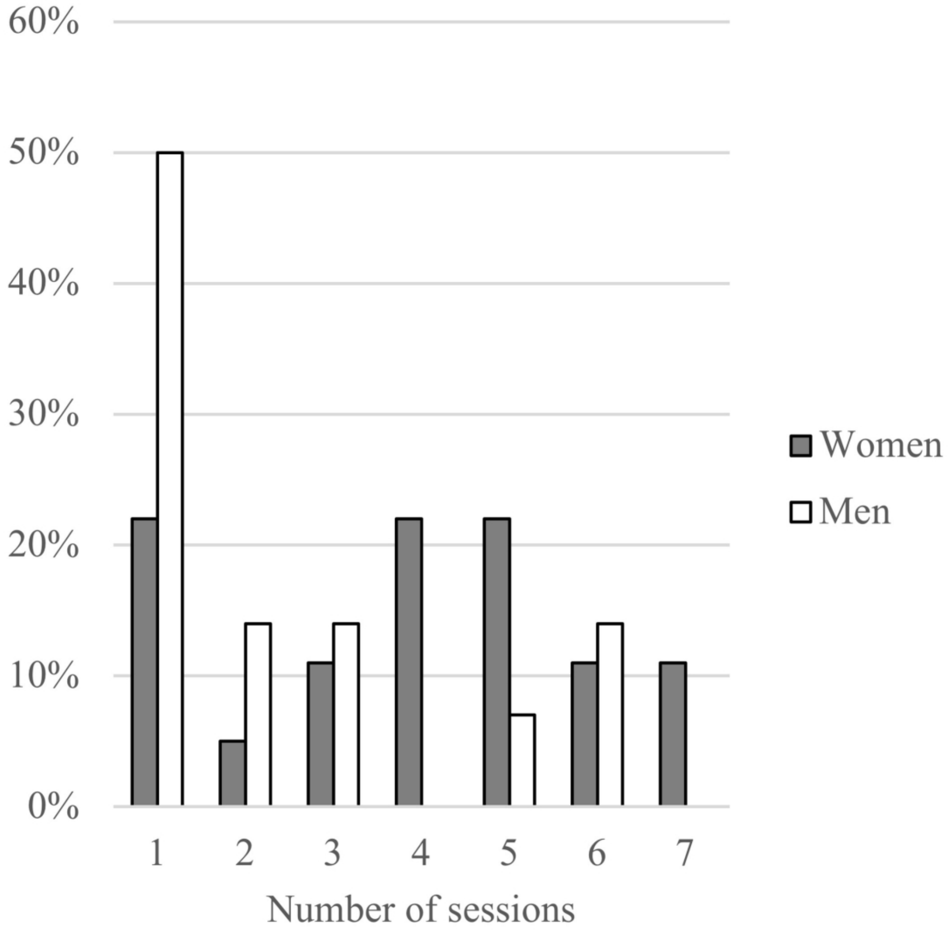

Gender participation rate in relation to total number of sessions during the intervention.

89x89mm (600 x 600 DPI)

Reporting checklist for qualitative study.

Based on the SRQR guidelines.

Instructions to authors

Complete this checklist by entering the page numbers from your manuscript where readers will find each of the items listed below.

Your article may not currently address all the items on the checklist. Please modify your text to include the missing information. If you are certain that an item does not apply, please write "n/a" and provide a short explanation.

Upload your completed checklist as an extra file when you submit to a journal.

In your methods section, say that you used the SRQR reporting guidelines, and cite them as:

O'Brien BC, Harris IB, Beckman TJ, Reed DA, Cook DA. Standards for reporting qualitative research: a synthesis of recommendations. Acad Med. 2014;89(9):1245-1251.

		Reporting Item	Page Number
	#1	Concise description of the nature and topic of the study identifying the study as qualitative or indicating the approach (e.g. ethnography, grounded theory) or data collection methods (e.g. interview, focus group) is recommended	3, 7
	#2	Summary of the key elements of the study using the abstract format of the intended publication; typically	3, 4

For peer review only

		includes background, purpose, methods, results and conclusions	
Problem formulation	#3	Description and significance of the problem / phenomenon studied: review of relevant theory and empirical work; problem statement	6, 7
Purpose or research question	#4	Purpose of the study and specific objectives or questions	7
Qualitative approach and research paradigm	#5	Qualitative approach (e.g. ethnography, grounded theory, case study, phenomenolgy, narrative research) and guiding theory if appropriate; identifying the research paradigm (e.g. postpositivist, constructivist / interpretivist) is also recommended; rationale. The rationale should briefly discuss the justification for choosing that theory, approach, method or technique rather than other options available; the assumptions and limitations implicit in those choices and how those choices influence study conclusions and transferability. As appropriate the rationale for several items might be discussed together.	3, 7—9 17—19
Researcher characteristics and reflexivity	#6	Researchers' characteristics that may influence the research, including personal attributes, qualifications / experience, relationship with participants, assumptions and / or presuppositions; potential or actual interaction between researchers' characteristics and the research	8, 9

For peer review only

		questions, approach, methods, results and / or transferability	
Context	#7	Setting / site and salient contextual factors; rationale	7, 8, 18
Sampling strategy	#8	How and why research participants, documents, or events were selected; criteria for deciding when no further sampling was necessary (e.g. sampling saturation); rationale	7, 8
Ethical issues pertaining to human subjects	#9	Documentation of approval by an appropriate ethics review board and participant consent, or explanation for lack thereof; other confidentiality and data security issues	9, 10
Data collection methods	#10	Types of data collected; details of data collection procedures including (as appropriate) start and stop dates of data collection and analysis, iterative process, triangulation of sources / methods, and modification of procedures in response to evolving study findings; rationale	7–9
Data collection instruments and technologies	#11	Description of instruments (e.g. interview guides, questionnaires) and devices (e.g. audio recorders) used for data collection; if / how the instruments(s) changed over the course of the study	8, 29
Units of study	#12	Number and relevant characteristics of participants, documents, or events included in the study; level of participation (could be reported in results)	3, 7,8, 28, figure 1

For peer review only

Data processing	#13	Methods for processing data prior to and during analysis, including transcription, data entry, data management and security, verification of data integrity, data coding, and anonymisation / deidentification of excerpts	8, 9
Data analysis	#14	Process by which inferences, themes, etc. were identified and developed, including the researchers involved in data analysis; usually references a specific paradigm or approach; rationale	9
Techniques to enhance trustworthiness	#15	Techniques to enhance trustworthiness and credibility of data analysis (e.g. member checking, audit trail, triangulation); rationale	8, 9
Syntheses and interpretation	#16	Main findings (e.g. interpretations, inferences, and themes); might include development of a theory or model, or integration with prior research or theory	10–16 18, 19
Links to empirical data	#17	Evidence (e.g. quotes, field notes, text excerpts, photographs) to substantiate analytic findings	10–16
Intergration with prior work, implications, transferability and contribution(s) to the field	#18	Short summary of main findings; explanation of how findings and conclusions connect to, support, elaborate on, or challenge conclusions of earlier scholarship; discussion of scope of application / generalizability; identification of unique contributions(s) to scholarship in a discipline or field	16, 17, 18, 19, 20

For peer review only

Limitations	#19	Trustworthiness and limitations of findings	5, 17–20
Conflicts of interest	#20	Potential sources of influence of perceived influence on study conduct and conclusions; how these were managed	17, 18, 20
Funding	#21	Sources of funding and other support; role of funders in data collection, interpretation and reporting	1

The SRQR checklist is distributed with permission of Wolters Kluwer © 2014 by the Association of American Medical Colleges. This checklist can be completed online using https://www.goodreports.org/, a tool made by the EQUATOR Network in collaboration with Penelope.ai

For peer review only

BMJ Open

Perceptions, experiences and barriers to lifestyle modifications in first-generation Middle Eastern immigrants to Sweden

Journal:	*BMJ Open*
Manuscript ID	bmjopen-2018-028076.R1
Article Type:	Original research
Date Submitted by the Author:	15-Jun-2019
Complete List of Authors:	Olaya-Contreras, Patricia; Sahlgrenska Academy, University of Gothenburg,, Institute of Health and Care Sciences<br>Balcker-Lundgren, Katarina; Center for Primary Health Care Research, Skåne University Hospital, Malmö, Sweden<br>Siddiqui , Faiza ; Lunds Universitet, Department of Clinical Sciences; Center for Primary Health Care Research, Skåne University Hospital, Malmö, Sweden<br>Bennet, Louise; Lund University, Center for Primary Health Care Research; Lund University, Malmö, Sweden
**Primary Subject Heading**:	Public health
Secondary Subject Heading:	Qualitative research
Keywords:	Type-2-Diabetes, lifestyle modifications, Middle-Eastern-immigrants, Qualitative Thematic Analysis, obesity-prevention, minority populations

SCHOLARONE™
Manuscripts

Manuscript title: Perceptions, experiences and barriers to lifestyle modifications in first-generation Middle Eastern immigrants to Sweden

Running title: Lifestyle modifications in Middle Eastern immigrants to Sweden

Word count: Manuscript 5989

Olaya-Contreras, P[1], Balcker, K[2], Siddiqui, F[2,3], Bennet, L[2,3]

[1]Institute of Health and Care Sciences, Sahlgrenska Academy, University of Gothenburg, Sweden.

[2] Center for Primary Health Care Research, Skåne University Hospital, Malmö, Sweden

[3] Department of Clinical Sciences, Lund University, Malmö, Sweden

Corresponding author

Patricia Olaya-Contreras

Institute of Health and Care Sciences, Sahlgrenska Academy, University of Gothenburg, Sweden.

Box 457, 405 30 Göteborg, Sweden

Patricia.olaya-contreras@gu.se

Phone number +46707 450383

For peer review only

For peer review only - http://bmjopen.bmj.com/site/about/guidelines.xhtml

Funding

This work was supported by grants from Lund University (ALF grants Dnr 20101641, 20101837, and 162641), Region Skåne (Dnr 226661 and 121811), the Swedish Society of Medicine (Dnr 97081 and 176831), the Crafoord Foundation (Dnr 20110719), the Swedish Research Council Linné grant to LUDC (Dnr 349-2006-237 and Dnr 349-2008-6589), Strategic Research area Exodiab (Dnr 2009-1039), Swedish Foundation for Strategic Research (Dnr IRC15-0067) and ANDIS (Dnr 825-2010-5983).

Competing interest

All authors have read and understood BMJ policy on declaration of interests and declare no competing interests.

For peer review only

Abstract

Objective: The prevalence of type 2 diabetes (T2D) among Iraqi immigrants to Sweden is high and partly related to sedentary physical activity and calorie dense food. The aim of the present study was to explore perceptions, experiences, and barriers concerning lifestyle modifications (LSM) in Iraqi immigrants to Sweden at risk for T2D.

Design: A qualitative thematic analysis was conducted on data collected from gender-specific focus group interviews which took place during a culturally adapted randomized controlled intervention study addressing motivation to lifestyle change, self-empowerment, behavioral modifications, and sociocultural barriers to lifestyle modification. Seven focus groups were held, with an interval of one to four weeks between January and May of 2015; each session lasted approximately 1.5 hours.

Setting: The city of Malmö, Sweden.

Participants: Out of 27 women and 23 men assigned to the intervention group, 19 women and 14 men attended at least one focus group session were included in the study.

Results: Participants expressed awareness of the content of healthy lifestyle practices. They also expressed numerous social and cultural barriers to LSM connected to irregular meals, overeating, food and drinking preferences and family expectations. Overeating was described as a consequence of social- and cultural norms and expectations, but also of poor mental wellbeing. Facilitators for reaching successful LSM were connected to family involvement and support.

Conclusion: Our study reports that facilitators for LSM are connected to presence of family support. Preventive actions addressing family involvement may benefit Middle Eastern immigrants at high risk for T2D to consider healthier lifestyles practices. Identification of sociocultural barriers and facilitators for LSM are crucial for successful health promotion in

For peer review only

For peer review only - http://bmjopen.bmj.com/site/about/guidelines.xhtml

minority populations at risk for T2D.

Keywords: Type 2 diabetes, obesity, prevention, Qualitative Thematic Analysis, lifestyle modifications, minority populations, Middle East

For peer review only - http://bmjopen.bmj.com/site/about/guidelines.xhtml

Article Summary

Strengths and limitations of the study

- A strength of this study is the use of focus group discussions, which encourage interaction between respondents to explore and clarify individual and shared perspectives on lifestyle modifications and views on the intervention.
- To address language barriers, a native Arabic speaker was present in all sessions who performed back- and forward translations when transcriptions were done.
- Perspectives on sociocultural barriers to lifestyle modification of younger, second generation Iraqi immigrant women and men are not represented in this study.
- Men had a lower attendance rate and barriers to lifestyle modification may be underreported.

For peer review only

Background

Political instability in the Middle East during recent decades has forced millions of people to flee their homelands and a large proportion of these migrants subsequently reach Europe. Today first generation immigrants from the Middle East constitute approximately 4% of the Swedish population, with the largest groups born in Iraq or Syria.[1]

Western versus non-western cultures often differ in terms of religion, traditions and social norms.[2] According to the World Health Organization (WHO), health is governed by so called 'social determinants' including environmental, socioeconomic factors and cultural norms influencing lifestyles.[3] Poor socioeconomic conditions contribute to higher diabetes incidence. For instance, non-western immigrants including Middle Eastern immigrants from Iraq to Denmark are often referred to live in socioeconomically deprived areas thus leading to higher morbidity and mortality rates.[4] In Sweden, refugees assigned to live in neighborhoods of high deprivation developed type 2 diabetes (T2D) to a higher extent compared to those assigned to a neighborhood of low deprivation.[5] Structural and sociocultural norms in immigrants and refugees influence lifestyle habits i.e. calorie dense foods and physical inactivity and are correlated with a high risk for T2D.[6-8]

In Sweden the prevalence of T2D among Middle Eastern immigrants is almost twice as high as compared to that of the native population.[9, 10] Despite more healthcare visits and the fact that they receive pharmacological treatment at an earlier stage, non-western immigrants have worse metabolic control, and develop diabetes complications at an earlier stage, compared to native Swedes.[11]

In ethnic minority populations, lifestyle interventions have been shown to be unsuccessful and individuals at risk do not reach target unless interventions are culturally adapted.[7] In Europe the few randomized culturally adapted interventions conducted on lifestyle mainly focused on

For peer review only - http://bmjopen.bmj.com/site/about/guidelines.xhtml

South-East Asian immigrants, such as the Innva-Diab Deplan study, and minority health study among Pakistani women,[12] the Physical activity and minority health study on Pakistani men in Norway, the Podosa trial in the UK,[13] and the SAHELI study on Surinamese immigrants in Netherlands.[14] However, religion, traditions and governing social norms as well as living context differs between South Asian and Middle Eastern populations, and also differs to the countries they have migrated to. Hence, this study was conducted to investigate the influence of sociocultural factors on lifestyle practices among Middle Eastern immigrants, specifically Iraqi immigrants, to increase the understanding of barriers and facilitators to lifestyle modifications (LSM) that Iraqi immigrants to Sweden have experienced.

Most qualitative studies have focused on studying the effect of sociocultural factors on LSM in ethnic minority populations from South-East Asia to Europe and the United States. The aim of the present study was accordingly to explore perceptions, experiences and barriers to LSM in immigrants from Iraq with overweight and at high risk of developing T2D.

Methods

The present study used a qualitative research design in the collection and analysis of data based on a culturally adapted intervention, the MEDIM cohort study 2010 to 2012.[9]

Participants and Public Involvement

The development of the research question. The majority of the immigrants to Sweden came from the Middle East, bringing with them another culture where lifestyle is governed by traditions and social norms that may differ to the Swedish culture. As immigrants, they are exposed to poor socioeconomic conditions and low levels of integration, which according to previous research create barriers to healthy lifestyles that can lead to a higher risk for T2D in this group when compared to native Swedish people. However, knowledge of what these

For peer review only - http://bmjopen.bmj.com/site/about/guidelines.xhtml

barriers look like is not well known or understood among immigrants from Iraq to Sweden.

Recruitment of participants: In the MEDIM study, approximately 45% out of the 1398 Iraqi-born participants were identified as being at high-risk for developing T2D during the next decade by being obese (BMI >28 kg/m^2) or having prediabetes. In 2015, these at risk individuals (N=636) fulfilled inclusion criteria and were invited to participate in this randomized intervention study (RCT) of four-month duration consisting of a culturally adapted LSM program that aimed to reduce diabetes risk.[15] Those diagnosed with diabetes, pregnant and/or poor physical or mental health were excluded from the study. A total of 96 first-generation Iraqi born immigrants signed informed consent to participate. Half of the participants were randomized to the intervention group (N=50) and half to the control group (N=46). The participants were not involved in the design, recruitment, or implementation of the RCT-study, nor in the design or evaluation of the intervention. None of the participants were known to the investigators prior to participation. The information analyzed in this study was gathered from the intervention group and the discussions from the group sessions.

Procedure

The intervention group were offered to participate in a total of seven gender specific group sessions, which were conducted in their native language. The sessions addressed awareness, motivation, taking action, relapse, review and feedback, setting goals and self-empowerment. The content of each group session is presented in Table 1, and was based on the evidence-based guidelines for prevention of T2D.[16] In accordance with the expected content of culturally adapted interventions,[6, 7] the sessions were 'culturally adapted' i.e. addressed knowledge gaps, gender specific groups and sociocultural barriers to lifestyle change.[15] Participants were also provided economic support for gym classes and gym clothing. The interview guide for the focus group sessions included structured and open ended questions to

For peer review only - http://bmjopen.bmj.com/site/about/guidelines.xhtml

explore a specific set of topics; first by asking a broader question addressing difficulties in reaching the goals, and subsequently asking more specific questions (Table 1). Participants individually answered the questions and were encouraged to interact with each other to further explore the views on the intervention. The topics in the interview guide are presented in detail in the Supplementary file. The interview guides were pilot tested prior to the intervention. An interdisciplinary team (specialist nurse in diabetes, health coach and Arabic and Swedish speaking translator) conducted the sessions. Each session took approximately 1 .5 hours, with intervals of 1–4 weeks, over a duration of four months.

All sessions were recorded. The interview recordings were subsequently translated by the official translator from Arabic to Swedish, and transcribed verbatim in Swedish. To check the translation from Arabic to Swedish, transcripts were checked against the recordings for accuracy, checking with the translator grammar and reviewing the translated data transcripts, and notes for data analyses by two of the authors (KB, LB) who are native Swedes. Thereafter, three of the authors (KB, LB, POC) were responsible for checking the accuracy of the translation of the cited quotations from Swedish to English. This ensured accuracy of the translation of the cited quotations i.e. confirming credibility.

Dissemination of findings: The findings will be disseminated by publications, interviews, lectures and media.

Data analysis

In analyzing the data gathered from the focus group interviews, an inductive thematic analysis was conducted, i.e., that the codes and themes derive from the content of the data themselves.[17] This method is an independent and reliable qualitative approach to analysis that provides a purely qualitative, detailed, and nuanced account of the data.[17, 18] The data were

For peer review only

For peer review only - http://bmjopen.bmj.com/site/about/guidelines.xhtml

organized to extract emergent patterns in the semantic content. All the authors read the transcribed data multiple times. A thematic codebook was developed to facilitate identification of main themes, thus allowing for the multiple coverage of possible topics. The first author made codes by marking important features in the transcribed text systematically across the entire data set and later collating data relevant to each code. Thereafter, the codes were organized into related areas to identify patterns and to construct descriptive themes. Then, a thematic map to make clear definitions and names for each theme was made. The authors met to discuss the codes, in order to identify new themes potentially converging or diverging, and further, to explore possible additional meanings in the content of participants' narratives.[17] This ensured that the complexity of experiences was reflected in the analysis (i.e. dependability, confirmability, credibility). A saturation point was attained when there was no additional information in terms of non-emergence of new codes of themes (Inductive thematic saturation).[19] A model exemplifying the analysis process, showing meanings and interpretations of Theme B is provided in Table 2. Major themes were defined as those responses/opinions voiced by more than half of the participants. NVivo Software program version 10 was used in the coding process.

Ethical considerations

All participants provided voluntary written informed consent prior to their participation in the present study. They were informed about the possibility to withdraw from the study without explanation and without consequences. The Ethical Review Board of Lund University, Sweden approved the study (approval no. 2011/88). All data collected were anonymized, and stored on a protected server only accessible by selected members of the research team.

Results

For peer review only - http://bmjopen.bmj.com/site/about/guidelines.xhtml

A total of 19 women and 14 men who participated in at least one group session were included in this study (Table 3). The majority had not completed secondary school (86%). Almost every second participant was unemployed (52%). Most were married with a family size of two to six persons. Female participants were, on average, eight years older than female non-participants (50.7 vs 42.6, *P*=0.049) whereas there were no significant age differences between male participants and non-participants (48.0 vs 46.4 y, *P*=0.747). There were no significant differences in BMI for male and female participants vs. non-participants (males 31.4 kg/m^2 vs 30.7 kg/m^2 *P*=0.699; females 30.8 kg/m^2 vs 31.2 kg/m^2 *P*=0.833). The main reason for non-participation was not receiving permission from work. Figure 1 presents participation rate in relation to total number of sessions during the intervention. Fifty percent of male participants participated in only one session however, 14% attended almost all sessions. Females had a more even attendance rate with 10-20% attending at least four sessions.

The findings are condensed into three major Themes: *A. Motivation to lifestyle modifications: Awareness of healthy diet habits and physical activities; B Perceptions, experiences and barriers towards healthier lifestyles; and C. Experienced obstacles and facilitators for maintaining lifestyle modifications following the intervention.*

A. Motivation to lifestyle modifications: Awareness of healthy diet habits and physical activities (sessions one and two)

The expression "healthy lifestyles" were understood as being physically active, i.e. doing sport/exercise, going to the gym regularly, eating vegetables and fruits, fibre rich- and

For peer review only - http://bmjopen.bmj.com/site/about/guidelines.xhtml

balanced diet:

> *To avoid sweets such as cookies, to drink water; To eat vegetables, as much as possible; To be physically active at least half an hour every day; To walk often; To reduce weight; You should sleep at least 7 hours; You should eat cooked food; One should not eat fried food; You should drink a lot of water and eat fish; You should eat regularly.*

They also expressed awareness of unhealthy lifestyles:

> *To consume too much red meat; to be obese or overweight; to drink soft drinks; to eat candies; to get stressed*

Physical inactivity was associated with illness and disease:

> *The reason why I have been physically active is to lose weight; I do not want a stroke or diabetes; Many in my family suffer from diabetes, so, I do not want to suffer from it.*

B. Perceptions, experiences and barriers towards healthier lifestyles (session three to five)

B.1 The challenge with regular meals

The participants described that they had difficulties having regular meals such as breakfast, lunch and dinner, often skipping the main meals, and commonly replacing meals with sweets. Among them, it was also common to have a late dinner, i.e. at night, sometimes they overate or on the contrary ate almost nothing.

Some participants explained that the reason was because their children ate at school, and

For peer review only

For peer review only - http://bmjopen.bmj.com/site/about/guidelines.xhtml

when they felt hungry they provided themselves with some snacks or fast food:

I do not usually eat so much lunch, I eat some chocolate when I get tired in the afternoon; I eat when I feel hungry, I do not think so much about what time to eat; I eat many meals often; At home, sometimes I go walking and eat, I have no specific time to eat.

B.2. Food preparation, taste preferences and portion size

Fried or sweet tasting foods were preferred and perceived as "*food with good smell and taste*", which they described as highly appreciated:

You do not feel good if you do not eat fried foods; I believe that I eat more fried products after walking because I need it

Some participants did not like the taste of water; instead they drank sodas and/or sweet juices. Others disliked the taste of foods boiled in water instead of being fried. A common statement was they neither consumed integral products with fiber rich flour nor low-calorie products. The ways to prepare foods or the kind of ingredients used were perceived as the most difficult to change:

I found it very difficult to change our diet, from the food that we like to eat, but I thought, I would be able to consume less sweet products

Participants liked to eat large portions. They had a habit of serving large portions, eating fried snacks, sweet beverages (sodas and tea with sugar), and candies. Some women believed that they were overweight because of how much they had eaten in each meal, and how much food they had eaten between meals, rather than because of how the food was prepared:

I realize that the problem, for me, it is the amount of food and the portion size.

For peer review only - http://bmjopen.bmj.com/site/about/guidelines.xhtml

B.3. Family traditions and expectations

A common statement was that the children decided what and where to eat, which explained some of the difficulties to change eating-habits. The mothers were eating fast food as their children did because of "*the need to follow family decisions*" and, in addition, the women explained their overeating because "*the boys need to eat enormous portions after exercising in the evening*". As a result of this, they prepared a lot of food and therefore they were serving themselves with big portions.

Nevertheless, some participants mentioned that after eating in excess they became sad or upset with themselves. Some participants drank soda on a daily basis (at least four cans per day) but were concerned with this and expressed that it needed to be stopped.

Family traditions and expectations influenced eating habits when celebrating or meeting relatives:

> *Yes, when I meet relatives, they invite me [to eat] all the time, and I cannot say no…; this is considered impolite not to eat the food when offered; It is hard to say no to good food that you get served; My family thinks I'm too small, but I'm overweight! They offer me different dishes and meals the whole time*

Some of the women expressed interest in exercising but they sometimes felt alone in this effort and did not always feel support from their family members for exercising. They also described they did not have enough free time for outdoors physical activity, or for going to the gym. Women commonly described that their time was mostly dedicated to household chores and time with the children:

> *I like to exercise but it's hard to find time when I have children; In my family, my*

For peer review only - http://bmjopen.bmj.com/site/about/guidelines.xhtml

children and my husband are not engaged in exercise and thus, I will be alone in my effort to exercise; I live in Sweden, I do not work, I take care of the household, children, shopping and cooking and have no time for sports; It´s difficult to get time for exercise, but I try to be physically active as often as it is possible.

<u>*B.4. Other barriers influencing motivation level*</u>

The participants expressed that they struggled to adapt to the cold and dark climate, which had a negative influence on outdoors physical activity, as one of the participants illustrated:

I get depressed when I eat less...; The weather is boring and the darkness too and I do not have so many social contacts; When I cook and the kids do not eat that much, I begin eating instead of exercising

It was stated that after some years without exercise, it was difficult to establish a routine and feel motivated to exercise regularly, and this feeling was worse when suffering from pain. One woman explained:

I have lived in Sweden for 8 years, I have four children, and I have not exercised in these years.

A male participant expressed:

Even though I'm on sick-leave, preferred to do other activities at home and do not have time to exercise.

C. Experienced obstacles and facilitators for maintaining lifestyle modifications following the intervention (session six to seven)

For peer review only - http://bmjopen.bmj.com/site/about/guidelines.xhtml

C.1. The paradox: raised awareness but still no lifestyle modifications

Despite the raised consciousness concerning the health benefits from changing dietary habits and increasing level of physical activity after the Intervention –especially the women – described that they continued eating sweets and pastries mainly when they were alone, and copiously, large portions when sharing with family and relatives. They found it difficult to lose weight, which they experienced, was related to social/family pressure. Most participants considered the goal of 10000 steps/day to be demanding because of lack of time to exercise and not feeling comfortable to go for a walk alone.

In fact, some of the women disliked go to the gym, they considered the gym as a “stuffy place”;

Further, they stated it was difficult to be alone there.

> *We would like to go to places only for women where we can exercise and do sports. These facilities need to be located near where we live; otherwise, it is not possible for us to go there.*

C.2. Changing lifestyle habits together

In some of the families, spouses were involved and supported the modifications implemented by their partner. In other families, the sessions influenced the whole family, i.e. controlling their children’s intake of sugar, and or preparing some dishes in a healthier way:

> *The whole family has changed dietary habits; My husband now eats only hard rye bread now and no white bread anymore; We eat a lot of vegetables, not like before; We bought whole grain rice and started using it instead; My husband has begun to check calories and ingredients in the food he buys, and now he is aware*

For peer review only - http://bmjopen.bmj.com/site/about/guidelines.xhtml

of the food we should not consume and those we should

Some participants described that they took regular walks together with family members, some started going to the gym:

I have started to walk a lot and be more physically active; I have started with water aerobics three times a week and working out twice a week; I also walk; I'm doing gymnastics, training 1 hour each day, 3 times a week and really, I feel better inside and outside; I had tried the treadmill and have trained with the machines in the gym. It is necessary to be strong to be able to lose weight and to get a healthier life

Two of the women involved their family members in domestic tasks, facilitating their possibility of having some extra time for PA:

My daughter and I walk together but my steps are more than hers are. I'm walking fast, I usually walk from 11 o´clock to 17; My son is practicing every day with me in the gym, so he helps me; I have lost 5-6 kilos, I think

During the intervention, some participants lost weight after increasing their everyday physical activity, despite some health problems, which is illustrated here:

Before, I weighed 77 kilos, and now I weigh 70 kilos; My goal was weight loss and although I have pain in my knee, I lost 10 kg; I have walked, and I need to continue walking or exercising to lose weight; I have focused on physical training and if I cannot walk, I exercise at home or go swimming

Discussion

Key findings

For peer review only - http://bmjopen.bmj.com/site/about/guidelines.xhtml

For peer review only

This is one of the first qualitative studies investigating self-perceived experiences, barriers and facilitators to LSM in Middle Eastern immigrants to Europe, representing a growing population at high risk for T2D. In this study of first-generation Iraqi immigrants to Sweden, family expectations and structural norms were identified as both strong barriers and facilitators for maintaining healthy lifestyle practices. Culturally adapted lifestyle intervention studies have been conducted previously in Europe; however, these studies are few and address the South Asian population.[12-14, 20] This study is novel in that it addresses the Middle Eastern immigrant population.

We have previously shown that this culturally adapted RCT study significantly reduced cardiometabolic risk in the intervention as compared to the control group. Further, this study showed positive impact of the intervention and group sessions not only on reported LSM but also on objectively assessed physical activity as well as metabolism.[15, 21]

Lifestyle challenges in the Middle Eastern immigrant population

The previous population based MEDIM study found a considerable proportion of the Iraqi immigrant population at very high risk for developing T2D within a decade.[9] The Middle Eastern population have other cultural aspects and traditions (such as language, food, religion and sociocultural norms) as compared to the South Asian population. Consequently, this RCT intervention study was initiated as a health initiative to prevent the Middle Eastern population from developing cardiometabolic disease; this study successfully reduced the cardiometabolic risk in these participants by combining different strategies.[15]

Different theoretical models link socioeconomic- and cultural factors with poor health. For

For peer review only - http://bmjopen.bmj.com/site/about/guidelines.xhtml

instance, in the 'Transitional Model', Schlossberg concludes that sets of factors related to contextual- and individual factors as well as environmental support seem to affect how one copes with transition and adaption to a new lifestyle.[22] Neighborhood deprivation predicts incidence of diabetes in refugees in Sweden,[5] which is in agreement with the WHO, stating that social determinants of health such as socioeconomic conditions, culture and social norms impacts health in chronic diseases morbidity.[3] Hence, LSM are fruitless unless environmental, social- and cultural barriers are identified and addressed.[5, 7] Furthermore, the term 'Social capital' has been suggested to be associated with health through several different pathways, including psychosocial mechanisms, norms regarding health related behaviors and access to health care, factors that at the population level are important for the maintenance of health and reduced mortality rates.[23] Middle Eastern immigrants to Sweden in general and Middle Eastern women, in particular, are exposed to all these different levels of social determinants, which is reflected by the fact that >60% of first-generation Iraqi immigrants in Malmö report low social capital (assessed as low social participation, low social anchorage, low instrumental- and low emotional support),[24] as well as high rates of morbidity in non-communicable diseases and poor mental health.[25] The low participation in this study could be a reflection of poor social capital, with low societal trust.

Perceptions, experiences and barriers towards healthier lifestyles

Although the awareness of the impact of healthy lifestyles on health, participants experienced difficulties to modify their lifestyles. The participants perceived that over time it was difficult to establish new dietary habits and become more physically active, which is consistent with studies showing that individuals living in socioeconomically vulnerable areas, challenges in

For peer review only - http://bmjopen.bmj.com/site/about/guidelines.xhtml

achieving modifications are often hindered by contextual and family influences in achieving lifestyle modifications.[26, 27]

The group sessions addressed knowledge gaps regarding how lifestyle influences the risk of developing T2D. However, the participants perceived as difficult to modify dietary preferences, especially when sharing with family and friends. The perceptions that food boiled in water rather than stir fried or unsweetened drinks do not taste good is a challenge to conquer, especially since eating patterns are commonly guided by family and children's expectations which in accordance with prior research.[26, 28-30]

Women commonly described feeling alone in their efforts to change their lifestyles. They felt that family expectations such as taking care of the children and managing the household had a negative influence on their ability to be physically active, which is consistent with what has been reported in other studies.[6-8, 18, 25-27, 29, 31, 32]

In the intervention, 10 000 steps per day was set as a daily goal, but it was perceived by most participants, as being unreachable. Beginning with lower step-goals can enhance adherence to the intervention in sedentary groups.[33] Thus, in this study, a reason for not complying with the intervention could be due to the perception that the goals were too difficult to reach.

Iraqi immigrants in Sweden and Pakistani immigrants in Norway show high prevalence of poor mental health and a strong connection between anxiety and depression with physical inactivity.[25, 34] Iraqi born participants in this study experienced that in order to cope with the dark cold climate and depressed mood they overate and preferred snacks instead of regular meals, which is in line with prior studies conducted among immigrant women at risk for T2D.[26, 27, 29, 31]

For peer review only - http://bmjopen.bmj.com/site/about/guidelines.xhtml

Facilitators for maintaining lifestyle modifications

Despite living in Sweden for more than two decades over 70% of Iraqi born immigrants in Malmö do not read or write in Swedish, and prefer traditional calorie dense foods,[35] reflecting low level of integration and acculturation.[24] Family traditions may impose cultural and social norms on each individual, being a barrier for successful LSM. On the other hand, our data indicate that strong family support being a corner stone in the Middle Eastern culture represents an important health promoting factor. The Transition Model highlights the importance of receiving support from both, family and environment to enhance adaptation, i.e. to cope with the intervention. Individuals move through the transition process, through a series of phases, i.e. "moving in," "moving through," and "moving out",[22] which may apply to our participants who need to cope, adapt, and make decisions across some cultural- and social situations to succeed with LSM.

The participants who managed to change their lifestyle described a profound support and involvement of their family members in this effort, i.e. motivating each other to maintain healthier habits. These findings correspond with previous research, in which, involving family in target interventions promotes healthier eating behaviors, increased physical activity as well as wellbeing among immigrant populations.[6, 26, 27, 29, 30, 36]

The availability for exercise centers, arenas and other physical environments are important contributors to lifestyles changes.[7, 26, 30, 37] In Malmö, there are several gyms for women and in this study, "Hälsans Hus" i.e. gym facilities only for women located in the nearby neighborhood was one of the physical activity centers women were referred to. Nevertheless,

For peer review only - http://bmjopen.bmj.com/site/about/guidelines.xhtml

the perceived lack of nearby gender specific gym facilities was a common reason among women to continue being physically inactive. Lack of time was another reason for not performing regular physical activity in this study. A previous study conducted in Sweden showed that Middle Eastern women wanted non- strenuous 'suitable and pleasant' exercise, i.e., cycling, swimming, and gymnastics and that it is important that the exercise is 'proper' i.e. not to exercise in front of the men.[32] Hence, close collaboration with the communities and families is mandatory to fully understand and adjust physical activity for different needs among non-westernized female immigrants, as confirmed by previous research.[7, 29, 32, 33, 37]

Strengths and limitations

Strengths of the study were the group discussions encouraging respondents to explore individual and shared perspectives and views on the intervention and focus attention on possibilities to improve healthy lifestyles. A native Arabic speaker was present in all sessions, and led back- and forward translations when transcriptions were performed, providing accuracy and credibility of the transcribed and analyzed information. In the last session – session #7, the participants provided feedback and addressed how they had handled obstacles and reached their goals throughout the intervention, thus assuring trustworthiness and confirmability of the information collected in this study. The participants did not know each other prior to the participation. Couples who had participated in the RCT study were randomized to either the intervention or control group,[15] thus, avoiding spread of information between the groups (in this qualitative study, only the intervention group was included). Some participants might have feared to express themselves openly in the group sessions. According to culturally adapted interventions,[7] this issue was addressed by having gender

For peer review only - http://bmjopen.bmj.com/site/about/guidelines.xhtml

specific groups to assure participants as far as possible could feel free to express their opinions. Although men participated to a lower extent than women, both men and women contributed to all themes as illustrated in Figure 1 where sessions 1 & 2 represented the theme "Motivation to lifestyle change"; sessions 3 to 5 "Perceptions, experiences and barriers towards healthier lifestyles"; sessions 6 & 7 "Experienced obstacles and facilitators for maintaining lifestyle modifications following the intervention". In accordance with the Ethics statement, participants could leave the study without any explanation, thus we do not have data on all reasons for leaving; however, a main reason for not participating was not receiving permission from work.

Our findings are limited to middle-aged first-generation Iraqi born immigrants rather than younger second generation participants. Further, ethnicity includes a variety of religious beliefs and family history therefore caution needs to be made when interpreting the data and when transferring our findings to other ethnical minority groups. However, the participants in this study have previously been considered representative for the Iraqi immigrant population living in Sweden.[9] Moreover, the findings of barriers to lifestyle change reported here correspond with previous culturally adapted intervention studies of other non-western immigrant populations as referred to above.

Conclusions

Identification of sociocultural barriers and facilitators for LSM in ethnic minority populations at increased risk of T2D are crucial for both health care professionals and society in order to successfully support populations at risk for T2D, and enable them to successfully reach healthier lifestyle practices. The present study highlighted that facilitators for LSM were

For peer review only

connected to profound family support. Tailoring interventions to include entire families, i.e., possibilities for childcare, and the involvement of spouse/other significant family or community member in the intervention could be successful strategies for increasing adherence to interventions/programs among Middle Eastern immigrants.

Barriers and facilitators for lifestyle change in ethnic minority populations are crucial for health care and society to acknowledge in the continued effort to promote healthier LSM in vulnerable populations at risk for non-communicable diseases.

For peer review only

For peer review only - http://bmjopen.bmj.com/site/about/guidelines.xhtml

References

1. Statistics Sweden. Available at http:www.scb.se.
2. Bhugra D, Becker MA. Migration, cultural bereavement and cultural identity. *World Psychiatry*. 2005; 4:18-24.
3. Marmot M, Friel S, Bell R, Houweling TA, Taylor S, Health CoSDo. Closing the gap in a generation: health equity through action on the social determinants of health. *Lancet*. 2008; 372:1661-9.
4. Jervelund SS, Malik S, Ahlmark N, Villadsen SF, Nielsen A, Vitus K. Morbidity, Self-Perceived Health and Mortality Among non-Western Immigrants and Their Descendants in Denmark in a Life Phase Perspective. *J Immigr Minor Health*. 2017; 19:448-76.
5. White JS, Hamad R, Li X, Basu S, Ohlsson H, Sundquist J, et al. Long-term effects of neighbourhood deprivation on diabetes risk: quasi-experimental evidence from a refugee dispersal policy in Sweden. *Lancet Diabetes Endocrinol*. 2016; 4:517-24.
6. Jaber LA, Pinelli NR, Brown MB, Funnell MM, Anderson R, Hammad A, et al. Feasibility of group lifestyle intervention for diabetes prevention in Arab Americans. *Diabetes Res Clin Pract*. 2011; 91:307-15.
7. Lirussi F. The global challenge of type 2 diabetes and the strategies for response in ethnic minority groups. *Diabetes Metab Res Rev*. 2010; 26:421-32.
8. Tiedje K, Wieland ML, Meiers SJ, Mohamed AA, Formea CM, Ridgeway JL, et al. A focus group study of healthy eating knowledge, practices, and barriers among adult and adolescent immigrants and refugees in the United States. *Int J Behav Nutr Phys Act*. 2014; 11:63.
9. Bennet L, Groop L, Lindblad U, Agardh CD, Franks PW. Ethnicity is an independent risk indicator when estimating diabetes risk with FINDRISC scores: a cross sectional study comparing immigrants from the Middle East and native Swedes. Prim Care Diabetes. 2014; 8:231-8.
10. Wändell PE, Carlsson A, Steiner KH. Prevalence of diabetes among immigrants in the Nordic countries. *Curr Diabetes Rev*. 2010; 6:126-33.
11. Rawshani A, Svensson AM, Rosengren A, Zethelius B, Eliasson B, Gudbjörnsdottir S. Impact of ethnicity on progress of glycaemic control in 131,935 newly diagnosed patients with type 2 diabetes: a nationwide observational study from the Swedish National Diabetes Register. *BMJ Open*. 2015; 5:e007599.
12. Telle-Hjellset V, Råberg Kjøllesdal MK, Bjørge B, Holmboe-Ottesen G, Wandel M, Birkeland KI, et al. The InnvaDiab-DE-PLAN study: a randomised controlled trial with a culturally adapted education programme improved the risk profile for type 2 diabetes in Pakistani immigrant women. *Br J Nutr*. 2013; 109:529-38.
13. Bhopal RS, Douglas A, Wallia S, Forbes JF, Lean ME, Gill JM, et al. Effect of a lifestyle intervention on weight change in south Asian individuals in the UK at high risk of type 2 diabetes: a family-cluster randomised controlled trial. *Lancet Diabetes Endocrinol*. 2014; 2:218-27.
14. Admiraal WM, Vlaar EM, Nierkens V, Holleman F, Middelkoop BJ, Stronks K, et al. Intensive lifestyle intervention in general practice to prevent type 2 diabetes among 18 to 60-year-old South Asians: 1-year effects on the weight status and metabolic profile of participants in a randomized controlled trial. *PLoS One*. 2013; 8:e68605.
15. Siddiqui F, Kurbasic A, Lindblad U, Nilsson PM, Bennet L. Effects of a culturally adapted lifestyle intervention on cardio-metabolic outcomes: a randomized controlled trial in Iraqi immigrants to Sweden at high risk for Type 2 diabetes. *Metabolism*. 2017; 66:1-13.
16. Paulweber B, Valensi P, Lindström J, Lalic NM, Greaves CJ, McKee M, et al. A European evidence-based guideline for the prevention of type 2 diabetes. *Horm Metab Res*. 2010; 42 Suppl 1:S3-36.
17. Braun V, Clarke V. What can "thematic analysis" offer health and wellbeing researchers? *Int J Qual Stud Health Well-being*. 2014; 9:26152.
18. Vaismoradi M, Turunen H, Bondas T. Content analysis and thematic analysis: Implications for conducting a qualitative descriptive study. *Nurs Health Sci*. 2013; 15:398-405.
19. Saunders B, Sim J, Kingstone T, Baker S, Waterfield J, Bartlam B, et al. Saturation in qualitative

For peer review only - http://bmjopen.bmj.com/site/about/guidelines.xhtml

research: exploring its conceptualization and operationalization. *Qual Quant*. 2018; 52:1893-907.
20. Andersen E, Burton NW, Anderssen SA. Physical activity levels six months after a randomised controlled physical activity intervention for Pakistani immigrant men living in Norway. *Int J Behav Nutr Phys Act*. 2012; 9:47.
21. Siddiqui F, Koivula RW, Kurbasic A, Lindblad U, Nilsson PM, Bennet L. Physical Activity in a Randomized Culturally Adapted Lifestyle Intervention. *Am J Prev Med.* 2018; 55:187-96.
22. Schlossberg N. A model for analyzing human adaption to transition. *Counseling Psychologist*. 1981; 9:2-18.
23. Kawachi I, Kennedy BP, Lochner K, Prothrow-Stith D. Social capital, income inequality, and mortality. *Am J Public Health*. 1997; 87:1491-8.
24. Bennet L, Lindström M. Self-rated health and social capital in Iraqi immigrants to Sweden: The MEDIM population-based study. *Scand J Public Health*. 2018; 46:194-203.
25. Siddiqui F, Lindblad U, Bennet L. Physical inactivity is strongly associated with anxiety and depression in Iraqi immigrants to Sweden: a cross-sectional study. *BMC Public Health*. 2014; 14:502.
26. Schneider S, Diehl K, Görig T, Schilling L, De Bock F, Hoffmann K, et al. Contextual influences on physical activity and eating habits -options for action on the community level. *BMC Public Health*. 2017; 17:760.
27. Pollard SL, Zachary DA, Wingert K, Booker SS, Surkan PJ. Family and Community Influences on Diabetes-Related Dietary Change in a Low-Income Urban Neighborhood. *Diabetes Educ*. 2014; 40:462-9.
28. Aaboud M, Aad G, Abbott B, Abdallah J, Abdinov O, Abeloos B, et al. Measurement of the [Formula: see text] and production cross sections in multilepton final states using 3.2 fb[Formula: see text] of [Formula: see text] collisions at [Formula: see text] = 13 TeV with the ATLAS detector. *Eur Phys J C Part Fields*. 2017; 77:40.
29. Wieland ML, Weis JA, Palmer T, Goodson M, Loth S, Omer F, et al. Physical activity and nutrition among immigrant and refugee women: a community-based participatory research approach. *Womens Health Issues*. 2012; 22:e225-32.
30. Everson-Hock ES, Johnson M, Jones R, Woods HB, Goyder E, Payne N, et al. Community-based dietary and physical activity interventions in low socioeconomic groups in the UK: a mixed methods systematic review. *Prev Med*. 2013; 56:265-72.
31. Sanchón-Macias MV, Bover-Bover A, Prieto-Salceda D, Paz-Zulueta M, Torres B, Gastaldo D. Determinants of Subjective Social Status and Health Among Latin American Women Immigrants in Spain: A Qualitative Approach. *J Immigr Minor Health*. 2016; 18:436-41.
32. Södergren M, Hylander I, Törnkvist L, Sundquist J, Sundquist K. Arranging appropriate activities immigrant women's ideas of enabling exercise. *Womens Health Issues*. 2008; 18:413-22.
33. Musto A, Jacobs K, Nash M, DelRossi G, Perry A. The effects of an incremental approach to 10,000 steps/day on metabolic syndrome components in sedentary overweight women. *J Phys Act Health*. 2010; 7:737-45.
34. Hjellset VT, Bjørge B, Eriksen HR, Høstmark AT. Risk factors for type 2 diabetes among female Pakistani immigrants: the InvaDiab-DEPLAN study on Pakistani immigrant women living in Oslo, Norway. *J Immigr Minor Health*. 2011; 13:101-10.
35. Siddiqui F, Winther V, Kurbasic A, Sonestedt E, Lundgren KB, Lindeberg S, et al. Changes in dietary intake following a culturally adapted lifestyle intervention among Iraqi immigrants to Sweden at high risk of type 2 diabetes: a randomised trial. *Public Health Nutr*. 2017; 20:2827-38.
36. Patel N, Ferrer HB, Tyrer F, Wray P, Farooqi A, Davies MJ, et al. Barriers and Facilitators to Healthy Lifestyle Changes in Minority Ethnic Populations in the UK: a Narrative Review. *J Racial Ethn Health Disparities*. 2017; 4:1107-19.
37. Cleland CL, Tully MA, Kee F, Cupples ME. The effectiveness of physical activity interventions in socio-economically disadvantaged communities: a systematic review. *Prev Med*. 2012; 54:371-80.

For peer review only - http://bmjopen.bmj.com/site/about/guidelines.xhtml

Table 1.

Session one (Awareness)	The group discussion addressed how unhealthy lifestyle habits predict risk of diabetes and cardiovascular disease, as well as discussions on nutritional contents of different foods.
Session two (Motivation/Getting started)	It focused on understanding the participant's current dietary and PA habits. The *Action Plan* was introduced as a tool that helped to formulate goals for lifestyle change and to support motivation for implementing lifestyle change, i.e. To write the personal goal and describing how a participant would reach it.
Session three (Taking Action /Maintaining the change)	It was designed to get started by putting dietary change into an Action Plan, by stimulating motivation for dietary change and PA and further by discussing how to achieve the postulated goals. The session also included discussions on common cooking methods for Iraqi food and how to make dishes healthier, i.e. consisting of less fat, sugar and more fibers.
Session four (Cooking session)	In the cooking session, participants were encouraged to bring the recipe of their favorite dish. This recipe was then modified by a professional chef at the cooking session to include healthier dishes including more fibers, less fat as well as less carbohydrates in the diet. The goal of this session was to highlight how cooking method, food preparation and portion size were vital components for effective lifestyle change.
Session five (Relapse – Maintaining the change)	To follow-up the action plan, and to identify barriers to its implementation, as well as experiences regarding social support, and family engagement toward the intervention. Facilitated rewrote action plan.
Session six (Review and feedback)	Participants had documented their everyday PA in a diary and this session included discussions on the final outcomes of lifestyle change, importance of maintaining lifestyle change, motivation to set new goals and discussions on external social support/network to reach those goals. The session also included feedback from the participants.
Session seven (Setting goals and Self-empowerment)	This session highlighted the importance of regularly setting new goals and reviewing these goals. This session also focused on empowering the participants and addressed how they had handled barriers and how they had evolved and reached their goals throughout the process.

Legend: Content of the discussions at each focus group session

For peer review only - http://bmjopen.bmj.com/site/about/guidelines.xhtml

Table 2.

THEME	SUBTHEME	Meanings and interpretations
Perceptions, experiences and further barriers for healthier lifestyles	Challenges with regular meals	• Loneliness at home affects willingness to eat regularly<br>• Eating fast food instead of regular meals<br>• Not perceiving the need for regular mealtimes and unestablished mealtime- schedules<br>• Eating repeatedly snacks and sweets<br>• Sometimes overeating or fasting
	Food preparation, taste preferences and portion size	• Preferences for eating food with "good smell and taste" over dishes prepared in a healthier way (boiled)<br>• Difficulties to introduce to the diet healthier food, healthier cooking, and low-calorie products<br>• Custom of eating large portions
	Family traditions and expectations	• The children decide what to eat in the family hindering a healthy diet<br>• Following family traditions when gathering to eat hindering the willingness to eat moderately<br>• Family resistance to exercise<br>• Challenges changing the role of the mother; limiting the mother´s time and possibilities to exercise
	Other barriers influencing motivation level	• Climate and related factors lessening outdoor physical activity and increasing overeating<br>• Depressed mood-isolation affecting willingness to participate in outdoor physical activities<br>• Difficulty to establish an exercise routine after many years of physical inactivity that worsens with concomitant pain

Legend: The analysis process, of meanings and interpretations of Theme B "Perceptions, experiences and barriers towards healthier lifestyles"

For peer review only

Table 3.

Session	Women	Men	Total/session
Session One: Awareness	14	9	23
Session Two: Motivation/getting started	15	7	22
Session Three: Taking Action /Maintaining the change	14	4	18
Session Four: Cooking session	9	7	16
Session Five: (Relapse – Maintaining the change)	9	3	12
Session Six: Review and feedback)	9	4	13
Session Seven: Setting goals and Self-empowerment	7	3	10

Legend: Group sessions with number of female and male participants throughout the intervention

For peer review only

Author contributions

POC: Wrote and revised the manuscript, analyzed the data and translated the material coming from the group discussions from Swedish to English.

KB: Participated in conducting the group sessions. Participated in data acquisition, reviewing the translated data transcripts, and notes from Sweden to English for data analyses, and data assessment. Contributed with interpretation of the data. Participated in writing and revising the manuscript.

FS: Participated in the design of the study and in conducting the group sessions. Participated in assessing data and interpretation of the data interpretation. Contributed to writing the manuscript.

LB: Designed and conducted the original study, contributed data acquisition, reviewing the translated data transcripts, and notes from Sweden to English for data analyses, data assessment and interpretation of the data. Contributed to writing and revised the manuscript.

Acknowledgements

We wish to acknowledge the invaluable contribution of the study participants. We are grateful to health coach Muna Mohammed, translator ImanYousif, study Nurses Saranda Muhaxheri, Josefin Goode Khan, and Asma Saleh, administrator Sonja Ruhnke and Ida Hagman. We are also grateful to Hälsans Hus, Rosengård, Abena, Viktväktarna, Arbetarnas Bildningsförbund (ABF) and Oatly. We thank Josephine T. V. Greenbrook for her help with the English language in the initial draft of the manuscript.

Data sharing Statement:

Data can be made available upon request to the corresponding author.

For peer review only

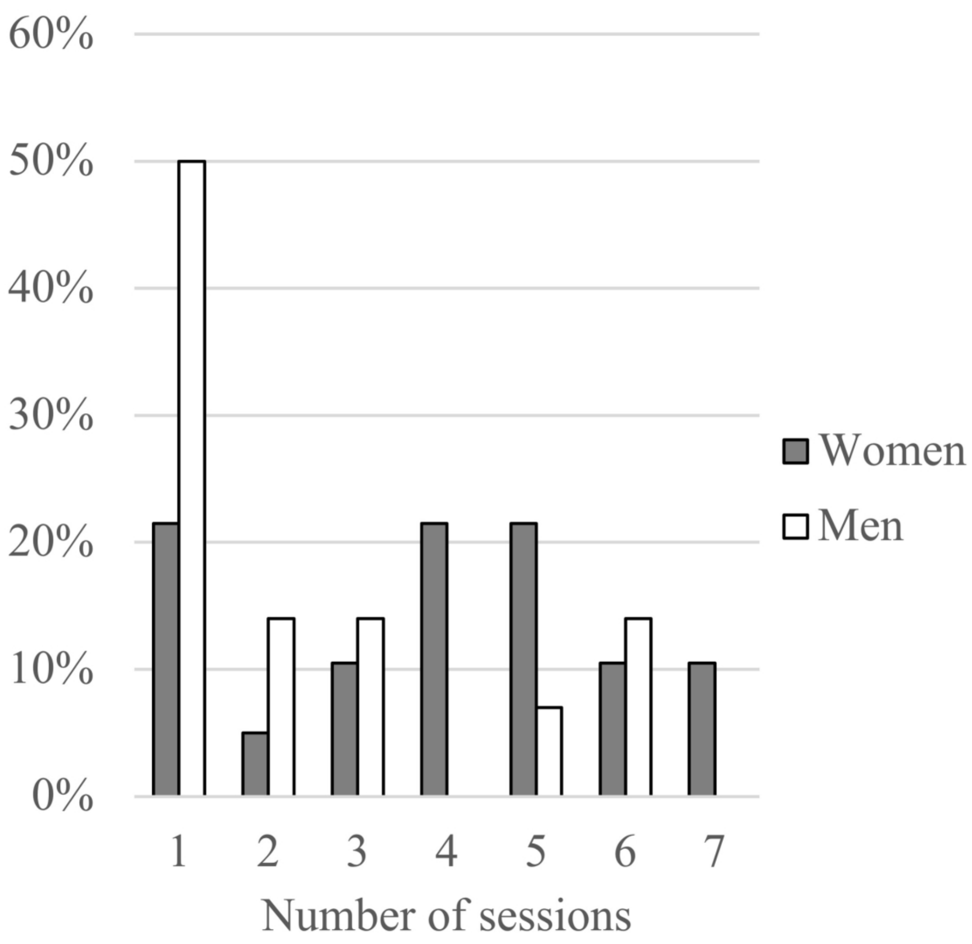

Gender participation rate in relation to total number of sessions during the intervention

89x89mm (600 x 600 DPI)

Group sessions the MEDIM Intervention study.

The intervention is modified from "The Image toolkit for prevention of type 2 diabetes in Europe: "Take action to prevent diabetes" http://www.image-project.eu/pdf/final_version_of_toolkit-perfect.pdf.

Questionnaires:

- Stage of change [2] or motivation level to enroll in an intervention study, to loose weight and increase their physical activity
- QALY EQ-5D [3]
- Questions about lifestyle habits (diet & exercise, psychological wellbeing)
- Background questions: socioeconomy, current medication and previous diseases, heredity regarding diabetes and cardiovascular diseases, acculturation
- Self-determination theory [4]
- SMART goals that form the basis for the Action Plan Work Sheet (will be developed)
- Physical activity diary
- Food diary

Additional comments:
Smartphone reminders as well as a "Support Line" via telephone.

Group meetings are recorded on tape to document what is said at meetings during discussion.

Session 1. To increase the awareness and knowledge of how lifestyle habits predicts risk of diabetes and cardiovascular disease. Identify health and unhealthy lifestyle habits

A. Identify present lifestyle habits:

1. **Let them fill out questionnaires regarding their present food and physical activity (PA) habits. Discuss their answers**

1. **To increase the awareness and knowledge of how lifestyle habits (food, PA, smoking) predicts risk of diabetes and cardiovascular disease.**
    a. Inform and discuss healthy level of PA and food intake.
    b. Discuss their answers from the questionnaires and ask the participants to identify healthy and unhealthy lifestyle habits regarding food and PA habits.

Session 2. "To stimulate motivation":

1. **To stimulate motivation**
    a. Let the participants describe lifestyle habits that they need to change. If they can't identify them, help them.
2. **Motivation**
    a. Identify and discuss barriers to lifestyle change
    b. Discuss strategies how to handle barriers
    c. Discuss the importance of social support
    d. Discuss pros and cons to lifestyle change ("motivation cross" used in Primary health care)
    e. Identify how important it is to them to change their lifestyle habits and let them fill out the form "Stage of change"
    f. Ask if
        i. They want to continue participating in the intervention
        ii. They want to think about it (participate in the same group again)
        iii. They don't want to continue (drop)
    g. Give them homework, to take a walk 15 minutes per day.
    h. Provide them with an Action plan work sheet and ask them to set a short term goal, when to do the activity, for how long and how often and what social support they can get. For example take a walk 15 minutes a day when the children are back from school so they can take care of their little 1 year old brother.

Session 3. "To get started/Taking Action"

Goals:

1. **To create an action plan**
2. **To handle and reduce the risk of relapse**
3. **Homework**

1. **To create an action plan**
    a. Ask the participants to identify lifestyle habits that they want to change
    b. Ask them to fill in the Stage of change form
    c. If they are ready for change ask them what changes they want to do.
    d. Introduce the SMART goal theory and practice to set short and long term goals (physical activity 150 min/week and 5% weight reduction from start). Make them create a ladder of short term goals that will ultimately lead to the big goal. The plan should also include social support they need to achieve and what coping strategies they will use if necessary.

e. Ask participants to write down their own action plan. (This action plan may be revised as they reach their short term goals. A diploma as they reach their short term goals, other ideas of rewards?)

2. To handle and reduce the risk of relapse

a. Discuss the behavioral change wheel, and explain that set backs are a part of the process and an opportunity to learn

b. Discuss and provide tools how to handle and deal with set backs

c. Discuss and provide tools how to handle negative thoughts and stress

d. Identify barriers to change and facilitators for lifestyle change. Identify things that are or are not helping them reaching their goals.

3. Homework

a. Practice action planning, identify negative thoughts and then counter with positive thoughts.

Session four: Cooking session

Session 5 to 7. "Maintaining lifestyle change"

Goals

1. Review the progress of lifestyle change

2. Review the behavioral change process

3. Repeat how to handle relapse

4. Rewrite the action plan and set new goals

1. Review the progress of lifestyle change

a. Go through the homework. What changes have they made? What barriers have they encountered? How did they deal with these barriers? What facilitated to reach goals?

b. Review the SMART goals and their Action plan work sheet. Focus on goals they have achieved. Emphasize the importance of self-monitoring and ask participants to evaluate their progress using their diaries, action plans and smart goals.

2. **Review the behavioral change process and the motivation for behavioral change.** Ask them if it is important to them to implement change and why. Let them fill in a stage of change form.

3. Repeat how to handle relapse.

a. Support successes and discuss setbacks and use them as educational opportunities. Have they made use of coping strategies? If, what has worked? What has not worked? Identify the obstacles that the participants experienced. Discuss in small groups of obstacles that were most relevant to them (emotional, cost-oriented, knowledge-related). In the group, discuss how to get past these barriers. Go through the large group what has been taken up in the small groups. Emphasize the importance of self-monitoring (for example the

use of the accelerometer, etc.). Ask the participants to reflect on what has worked and what has not worked. Make sure the focus is on reaching milestones so that it is easier to build self-confidence and opportunities to achieve success. Ask participants to reflect on their expectations of what to achieve are realistic.

b. Illustrate the importance of periodically review goals and how they evolved. Which goals are they most pleased to have reached? Have there been any unexpected positive changes? Ask participants to identify ways that they can reward themselves.

c. Ask participants to identify positive and negative sources of social support that they had experienced. Did the social support work that they put into their action plan? If not, why? Ask the group to develop more ideas for how social support can act.

4. Rewrite the action plan and set new goals

For peer review only

Reporting checklist for qualitative study.

Based on the SRQR guidelines.

Instructions to authors

Complete this checklist by entering the page numbers from your manuscript where readers will find each of the items listed below.

Your article may not currently address all the items on the checklist. Please modify your text to include the missing information. If you are certain that an item does not apply, please write "n/a" and provide a short explanation.

Upload your completed checklist as an extra file when you submit to a journal.

In your methods section, say that you used the SRQR reporting guidelines, and cite them as:

O'Brien BC, Harris IB, Beckman TJ, Reed DA, Cook DA. Standards for reporting qualitative research: a synthesis of recommendations. Acad Med. 2014;89(9):1245-1251.

		Reporting Item	Page Number
	#1	Concise description of the nature and topic of the study identifying the study as qualitative or indicating the approach (e.g. ethnography, grounded theory) or data collection methods (e.g. interview, focus group) is recommended	3, 7
	#2	Summary of the key elements of the study using the abstract format of the intended publication; typically includes background, purpose, methods, results and conclusions	3, 4
Problem formulation	#3	Description and significance of the problem / phenomenon studied: review of relevant theory and empirical work; problem statement	6, 7
Purpose or research question	#4	Purpose of the study and specific objectives or questions	7
Qualitative approach and research paradigm	#5	Qualitative approach (e.g. ethnography, grounded theory, case study, phenomenolgy, narrative research) and guiding theory if appropriate;	3, 7—10 18—19

For peer review only

		identifying the research paradigm (e.g. postpositivist, constructivist / interpretivist) is also recommended; rationale. The rationale should briefly discuss the justification for choosing that theory, approach, method or technique rather than other options available; the assumptions and limitations implicit in those choices and how those choices influence study conclusions and transferability. As appropriate the rationale for several items might be discussed together.	20—22<br>23
Researcher characteristics and reflexivity	#6	Researchers' characteristics that may influence the research, including personal attributes, qualifications / experience, relationship with participants, assumptions and / or presuppositions; potential or actual interaction between researchers' characteristics and the research questions, approach, methods, results and / or transferability	7—10<br>22
Context	#7	Setting / site and salient contextual factors; rationale	3, 6—8,<br>18—19,21
Sampling strategy	#8	How and why research participants, documents, or events were selected; criteria for deciding when no further sampling was necessary (e.g. sampling saturation); rationale	7—10
Ethical issues pertaining to human subjects	#9	Documentation of approval by an appropriate ethics review board and participant consent, or explanation for lack thereof; other confidentiality and data security issues	7, 10
Data collection methods	#10	Types of data collected; details of data collection procedures including (as appropriate) start and stop dates of data collection and analysis, iterative process, triangulation of sources / methods, and modification of procedures in response to evolving study findings; rationale	3, 7—10
Data collection instruments and technologies	#11	Description of instruments (e.g. interview guides, questionnaires) and devices (e.g. audio recorders)	8-9, 26 Table1

For peer review only

		used for data collection; if / how the instruments(s) changed over the course of the study	Supplementary file
Units of study	#12	Number and relevant characteristics of participants, documents, or events included in the study; level of participation (could be reported in results)	3,5, 7,8, 10-11, 22, 28, figure 1, Table 3
Data processing	#13	Methods for processing data prior to and during analysis, including transcription, data entry, data management and security, verification of data integrity, data coding, and anonymisation / deidentification of excerpts	8—10
Data analysis	#14	Process by which inferences, themes, etc. were identified and developed, including the researchers involved in data analysis; usually references a specific paradigm or approach; rationale	9—10, Table 2
Techniques to enhance trustworthiness	#15	Techniques to enhance trustworthiness and credibility of data analysis (e.g. member checking, audit trail, triangulation); rationale	9—10
Syntheses and interpretation	#16	Main findings (e.g. interpretations, inferences, and themes); might include development of a theory or model, or integration with prior research or theory	10—17 20—21
Links to empirical data	#17	Evidence (e.g. quotes, field notes, text excerpts, photographs) to substantiate analytic findings	10—17, 27
Intergration with prior work, implications, transferability and contribution(s) to the field	#18	Short summary of main findings; explanation of how findings and conclusions connect to, support, elaborate on, or challenge conclusions of earlier scholarship; discussion of scope of application / generalizability; identification of unique contributions(s) to scholarship in a discipline or field	3, 17—19, 20—21 21—23
Limitations	#19	Trustworthiness and limitations of findings	5, 8—10, 19,22—23

For peer review

Conflicts of interest	#20	Potential sources of influence of perceived influence on study conduct and conclusions; how these were managed	8–9, 21–23
Funding	#21	Sources of funding and other support; role of funders in data collection, interpretation and reporting	2

The SRQR checklist is distributed with permission of Wolters Kluwer © 2014 by the Association of American Medical Colleges. This checklist can be completed online using https://www.goodreports.org/, a tool made by the EQUATOR Network in collaboration with Penelope.ai

For peer review only

BMJ Open

Perceptions, experiences and barriers to lifestyle modifications in first-generation Middle Eastern immigrants to Sweden – A qualitative study

Journal:	*BMJ Open*
Manuscript ID	bmjopen-2018-028076.R2
Article Type:	Original research
Date Submitted by the Author:	09-Sep-2019
Complete List of Authors:	Olaya-Contreras, Patricia; Sahlgrenska Academy, University of Gothenburg,, Institute of Health and Care Sciences<br>Balcker-Lundgren, Katarina; Center for Primary Health Care Research, Skåne University Hospital, Malmö, Sweden<br>Siddiqui , Faiza ; Lunds Universitet, Department of Clinical Sciences; Center for Primary Health Care Research, Skåne University Hospital, Malmö, Sweden<br>Bennet, Louise; Lund University, Center for Primary Health Care Research; Lund University, Malmö, Sweden
**Primary Subject Heading**:	Public health
Secondary Subject Heading:	Qualitative research, Diabetes and endocrinology
Keywords:	Type-2-Diabetes, lifestyle modifications, Middle-Eastern-immigrants, Qualitative Thematic Analysis, obesity-prevention, minority populations

SCHOLARONE™
Manuscripts

Manuscript title: Perceptions, experiences and barriers to lifestyle modifications in first-generation Middle Eastern immigrants to Sweden – A qualitative study

Running title: Lifestyle modifications in Middle Eastern immigrants to Sweden

Word count: Manuscript 5989

Olaya-Contreras, P[1], Balcker, K[2], Siddiqui, F[2,3], Bennet, L[2,3]

[1]Institute of Health and Care Sciences, Sahlgrenska Academy, University of Gothenburg, Sweden.

[2] Center for Primary Health Care Research, Skåne University Hospital, Malmö, Sweden

[3] Department of Clinical Sciences, Lund University, Malmö, Sweden

Corresponding author

Patricia Olaya-Contreras

Institute of Health and Care Sciences, Sahlgrenska Academy, University of Gothenburg, Sweden.

Box 457, 405 30 Göteborg, Sweden

Email: Patricia.olaya-contreras@gu.se

Phone number +46707 450383

For peer review only

Funding

This work was supported by grants from Lund University (ALF grants Dnr 20101641, 20101837, and 162641), Region Skåne (Dnr 226661 and 121811), the Swedish Society of Medicine (Dnr 97081 and 176831), the Crafoord Foundation (Dnr 20110719), the Swedish Research Council Linné grant to LUDC (Dnr 349-2006-237 and Dnr 349-2008-6589), Strategic Research area Exodiab (Dnr 2009-1039), Swedish Foundation for Strategic Research (Dnr IRC15-0067) and ANDIS (Dnr 825-2010-5983).

Competing interest

All authors have read and understood BMJ policy on competing interests. All authors declare none competing interests.

For peer review only

For peer review only - http://bmjopen.bmj.com/site/about/guidelines.xhtml

Abstract

Objective: The prevalence of type 2 diabetes (T2D) among Iraqi immigrants to Sweden is high and partly related to sedentary physical activity and calorie dense food. The aim of the present study was to explore perceptions, experiences, and barriers concerning lifestyle modifications (LSM) in Iraqi immigrants to Sweden at risk for T2D.

Design: A qualitative thematic analysis was conducted on data collected from gender-specific focus group interviews which took place during a culturally adapted randomized controlled intervention study addressing motivation to lifestyle change, self-empowerment, behavioral modifications, and sociocultural barriers to lifestyle modification. Seven focus groups were held, with an interval of one to four weeks between January and May of 2015; each session lasted approximately 1.5 hours.

Setting: The city of Malmö, Sweden.

Participants: Out of 27 women and 23 men assigned to the intervention group, 19 women and 14 men attended at least one focus group session were included in the study.

Results: Participants expressed awareness of the content of healthy lifestyle practices. They also expressed numerous social and cultural barriers to LSM connected to irregular meals, overeating, food and drinking preferences and family expectations. Overeating was described as a consequence of social- and cultural norms and expectations, but also of poor mental wellbeing. Facilitators for reaching successful LSM were connected to family involvement and support.

Conclusion: Our study reports that facilitators for LSM are connected to presence of family support. Preventive actions addressing family involvement may benefit Middle Eastern immigrants at high risk for T2D to consider healthier lifestyles practices. Identification of sociocultural barriers and facilitators for LSM are crucial for successful health promotion in

For peer review only

For peer review only - http://bmjopen.bmj.com/site/about/guidelines.xhtml

minority populations at risk for T2D.

Keywords: Type 2 diabetes, obesity, prevention, Qualitative Thematic Analysis, lifestyle modifications, minority populations, Middle East

For peer review only - http://bmjopen.bmj.com/site/about/guidelines.xhtml

Article Summary

Strengths and limitations of the study

- A strength of this study is the use of focus group discussions, which encourage interaction between respondents to explore and clarify individual and shared perspectives on lifestyle modifications and views on the intervention.
- To address language barriers, a native Arabic speaker was present in all sessions who performed back- and forward translations when transcriptions were done.
- Perspectives on sociocultural barriers to lifestyle modification of younger, second generation Iraqi immigrant women and men are not represented in this study.
- Men had a lower attendance rate and barriers to lifestyle modification may be underreported.

For peer review only

For peer review only - http://bmjopen.bmj.com/site/about/guidelines.xhtml

Background

Political instability in the Middle East during recent decades has forced millions of people to flee their homelands and a large proportion of these migrants subsequently reach Europe. Today first generation immigrants from the Middle East constitute approximately 4% of the Swedish population, with the largest groups born in Iraq or Syria.[1]

Western versus non-western cultures often differ in terms of religion, traditions and social norms.[2] According to the World Health Organization (WHO), health is governed by so called 'social determinants' including environmental, socioeconomic factors and cultural norms influencing lifestyles.[3] Poor socioeconomic conditions contribute to higher diabetes incidence. For instance, non-western immigrants including Middle Eastern immigrants from Iraq to Denmark are often referred to live in socioeconomically deprived areas thus leading to higher morbidity and mortality rates.[4] In Sweden, refugees assigned to live in neighborhoods of high deprivation developed type 2 diabetes (T2D) to a higher extent compared to those assigned to a neighborhood of low deprivation.[5] Structural and sociocultural norms in immigrants and refugees influence lifestyle habits i.e. calorie dense foods and physical inactivity and are correlated with a high risk for T2D.[6-8]

In Sweden the prevalence of T2D among Middle Eastern immigrants is almost twice as high as compared to that of the native population.[9, 10] Despite more healthcare visits and the fact that they receive pharmacological treatment at an earlier stage, non-western immigrants have worse metabolic control, and develop diabetes complications at an earlier stage, compared to native Swedes.[11]

In ethnic minority populations, lifestyle interventions have been shown to be unsuccessful and individuals at risk do not reach target unless interventions are culturally adapted.[7] In Europe the few randomized culturally adapted interventions conducted on lifestyle mainly focused on

For peer review only - http://bmjopen.bmj.com/site/about/guidelines.xhtml

South-East Asian immigrants, such as the Innva-Diab Deplan study, and minority health study among Pakistani women,[12] the Physical activity and minority health study on Pakistani men in Norway, the Podosa trial in the UK,[13] and the SAHELI study on Surinamese immigrants in Netherlands.[14] However, religion, traditions and governing social norms as well as living context differs between South Asian and Middle Eastern populations, and also differs to the countries they have migrated to. Hence, this study was conducted to investigate the influence of sociocultural factors on lifestyle practices among Middle Eastern immigrants, specifically Iraqi immigrants, to increase the understanding of barriers and facilitators to lifestyle modifications (LSM) that Iraqi immigrants to Sweden have experienced.

Most qualitative studies have focused on studying the effect of sociocultural factors on LSM in ethnic minority populations from South-East Asia to Europe and the United States. The aim of the present study was accordingly to explore perceptions, experiences and barriers to LSM in immigrants from Iraq with overweight and at high risk of developing T2D.

Methods

The present study used a qualitative research design in the collection and analysis of data based on a culturally adapted intervention, the MEDIM cohort study 2010 to 2012.[9]

Recruitment of participants: In the MEDIM study, approximately 45% out of the 1398 Iraqi-born participants were identified as being at high-risk for developing T2D during the next decade by being obese (BMI >28 kg/m^2) or having prediabetes. In 2015, these at risk individuals (N=636) fulfilled inclusion criteria and were invited to participate in this randomized intervention study (RCT) of four-month duration consisting of a culturally adapted LSM program that aimed to reduce diabetes risk.[15] Those diagnosed with diabetes, pregnant and/or poor physical or mental health were excluded from the study. A total of 96

For peer review only - http://bmjopen.bmj.com/site/about/guidelines.xhtml

first-generation Iraqi born immigrants signed informed consent to participate. Half of the participants were randomized to the intervention group (N=50) and half to the control group (N=46). None of the participants were known to the investigators prior to participation. The information analyzed in this study was gathered from the intervention group and the discussions from the group sessions.

Procedure and data collection

The intervention group were offered to participate in a total of seven gender specific group sessions, which were conducted in their native language. The sessions addressed awareness, motivation, taking action, relapse, review and feedback, setting goals and self-empowerment. The content of each group session is presented in Table 1, and was based on the evidence-based guidelines for prevention of T2D.[16] In accordance with the expected content of culturally adapted interventions,[6, 7] the sessions were 'culturally adapted' i.e. addressed knowledge gaps, gender specific groups and sociocultural barriers to lifestyle change.[15] Participants were also provided economic support for gym classes and gym clothing. The interview guide for the focus group sessions included structured and open-ended questions to explore a specific set of topics; first by asking a broader question addressing difficulties in reaching the goals, and subsequently asking more specific questions (Table 1). Participants individually answered the questions and were encouraged to interact with each other to further explore the views on the intervention. The topics in the interview guide are presented in detail in the Supplementary file. The interview guides were pilot tested prior to the intervention. An interdisciplinary team (specialist nurse in diabetes, health coach and Arabic and Swedish speaking translator) conducted the sessions. Each session took approximately 1 .5 hours, with intervals of 1–4 weeks, over a duration of four months.

All sessions were recorded. The interview recordings were subsequently translated by the

For peer review only - http://bmjopen.bmj.com/site/about/guidelines.xhtml

official translator from Arabic to Swedish, and transcribed verbatim in Swedish. To check the translation from Arabic to Swedish, transcripts were checked against the recordings for accuracy, checking with the translator grammar and reviewing the translated data transcripts, and notes for data analyses by two of the authors (KB, LB) who are native Swedes. Thereafter, three of the authors (KB, LB, POC) were responsible for checking the accuracy of the translation of the cited quotations from Swedish to English. This ensured accuracy of the translation of the cited quotations i.e. confirming credibility.

Data analysis

In analyzing the data gathered from the focus group interviews, an inductive thematic analysis was conducted, i.e., that the codes and themes derive from the content of the data themselves.[17] This method is an independent and reliable qualitative approach to analysis that provides a purely qualitative, detailed, and nuanced account of the data.[17, 18] The data were organized to extract emergent patterns in the semantic content. All the authors read the transcribed data multiple times. A thematic codebook was developed to facilitate identification of main themes, thus allowing for the multiple coverage of possible topics. The first author made codes by marking important features in the transcribed text systematically across the entire data set and later collating data relevant to each code. Thereafter, the codes were organized into related areas to identify patterns and to construct descriptive themes. Then, a thematic map to make clear definitions and names for each theme was made. The authors met to discuss the codes, in order to identify new themes potentially converging or diverging, and further, to explore possible additional meanings in the content of participants' narratives.[17] This ensured that the complexity of experiences was reflected in the analysis (i.e. dependability, confirmability, credibility). A saturation point was attained when there was no

For peer review only - http://bmjopen.bmj.com/site/about/guidelines.xhtml

additional information in terms of non-emergence of new codes of themes (Inductive thematic saturation).[19] A model exemplifying the analysis process, showing meanings and interpretations of Theme B is provided in Table 2. Major themes were defined as those responses/opinions voiced by more than half of the participants. NVivo Software program version 10 was used in the coding process.

Ethical considerations

All participants provided voluntary written informed consent prior to their participation in the present study. They were informed about the possibility to withdraw from the study without explanation and without consequences. The Ethical Review Board of Lund University, Sweden approved the study (approval no. 2011/88). All data collected were anonymized, and stored on a protected server only accessible by selected members of the research team.

***Patient and Public Involvement statement*:** The participants were not involved in the design or planning of the study. The findings will be disseminated to the public through the present scientific publication in BMJ Open, interviews, lectures and media.

Results

A total of 19 women and 14 men who participated in at least one group session were included in this study (Table 3). The majority had not completed secondary school (86%). Almost every second participant was unemployed (52%). Most were married with a family size of two to six persons. Female participants were, on average, eight years older than female non-participants (50.7 vs 42.6, P=0.049) whereas there were no significant age differences between male participants and non-participants (48.0 vs 46.4 y, P=0.747). There were no significant differences in BMI for male and female participants vs. non-participants (males

For peer review only - http://bmjopen.bmj.com/site/about/guidelines.xhtml

31.4 kg/m^2 vs 30.7 kg/m^2 P=0.699; females 30.8 kg/m^2 vs 31.2 kg/m^2 P=0.833). The main reason for non-participation was not receiving permission from work. Figure 1 presents participation rate in relation to total number of sessions during the intervention. Fifty percent of male participants participated in only one session however, 14% attended almost all sessions. Females had a more even attendance rate with 10-20% attending at least four sessions.

The findings are condensed into three major Themes: *A. Motivation to lifestyle modifications: Awareness of healthy diet habits and physical activities; B Perceptions, experiences and barriers towards healthier lifestyles; and C. Experienced obstacles and facilitators for maintaining lifestyle modifications following the intervention.*

A. Motivation to lifestyle modifications: Awareness of healthy diet habits and physical activities (sessions one and two)

The expression "healthy lifestyles" were understood as being physically active, i.e. doing sport/exercise, going to the gym regularly, eating vegetables and fruits, fibre rich- and balanced diet:

> *To avoid sweets such as cookies, to drink water; To eat vegetables, as much as possible; To be physically active at least half an hour every day; To walk often; To reduce weight; You should sleep at least 7 hours; You should eat cooked food; One should not eat fried food; You should drink a lot of water and eat fish; You should eat regularly.*

For peer review only - http://bmjopen.bmj.com/site/about/guidelines.xhtml

They also expressed awareness of unhealthy lifestyles:

To consume too much red meat; to be obese or overweight; to drink soft drinks; to eat candies; to get stressed

Physical inactivity was associated with illness and disease:

The reason why I have been physically active is to lose weight; I do not want a stroke or diabetes; Many in my family suffer from diabetes, so, I do not want to suffer from it.

B. Perceptions, experiences and barriers towards healthier lifestyles (session three to five)

B.1 The challenge with regular meals

The participants described that they had difficulties having regular meals such as breakfast, lunch and dinner, often skipping the main meals, and commonly replacing meals with sweets. Among them, it was also common to have a late dinner, i.e. at night, sometimes they overate or on the contrary ate almost nothing.

Some participants explained that the reason was because their children ate at school, and when they felt hungry they provided themselves with some snacks or fast food:

I do not usually eat so much lunch, I eat some chocolate when I get tired in the afternoon; I eat when I feel hungry, I do not think so much about what time to eat; I eat many meals often; At home, sometimes I go walking and eat, I have no specific time to eat.

B.2. Food preparation, taste preferences and portion size

Fried or sweet tasting foods were preferred and perceived as "*food with good smell and taste*",

For peer review only - http://bmjopen.bmj.com/site/about/guidelines.xhtml

which they described as highly appreciated:

> *You do not feel good if you do not eat fried foods; I believe that I eat more fried products after walking because I need it*

Some participants did not like the taste of water; instead they drank sodas and/or sweet juices. Others disliked the taste of foods boiled in water instead of being fried. A common statement was they neither consumed integral products with fiber rich flour nor low-calorie products. The ways to prepare foods or the kind of ingredients used were perceived as the most difficult to change:

> *I found it very difficult to change our diet, from the food that we like to eat, but I thought, I would be able to consume less sweet products*

Participants liked to eat large portions. They had a habit of serving large portions, eating fried snacks, sweet beverages (sodas and tea with sugar), and candies. Some women believed that they were overweight because of how much they had eaten in each meal, and how much food they had eaten between meals, rather than because of how the food was prepared:

> *I realize that the problem, for me, it is the amount of food and the portion size.*

B.3. Family traditions and expectations

A common statement was that the children decided what and where to eat, which explained some of the difficulties to change eating-habits. The mothers were eating fast food as their children did because of "*the need to follow family decisions*" and, in addition, the women explained their overeating because "*the boys need to eat enormous portions after exercising in the evening"*. As a result of this, they prepared a lot of food and therefore they were serving themselves with big portions.

For peer review only

Nevertheless, some participants mentioned that after eating in excess they became sad or upset with themselves. Some participants drank soda on a daily basis (at least four cans per day) but were concerned with this and expressed that it needed to be stopped.

Family traditions and expectations influenced eating habits when celebrating or meeting relatives:

> *Yes, when I meet relatives, they invite me [to eat] all the time, and I cannot say no…; this is considered impolite not to eat the food when offered; It is hard to say no to good food that you get served; My family thinks I'm too small, but I'm overweight! They offer me different dishes and meals the whole time*

Some of the women expressed interest in exercising but they sometimes felt alone in this effort and did not always feel support from their family members for exercising. They also described they did not have enough free time for outdoors physical activity, or for going to the gym. Women commonly described that their time was mostly dedicated to household chores and time with the children:

> *I like to exercise but it's hard to find time when I have children; In my family, my children and my husband are not engaged in exercise and thus, I will be alone in my effort to exercise; I live in Sweden, I do not work, I take care of the household, children, shopping and cooking and have no time for sports; It´s difficult to get time for exercise, but I try to be physically active as often as it is possible.*

B.4. Other barriers influencing motivation level

The participants expressed that they struggled to adapt to the cold and dark climate, which

For peer review only - http://bmjopen.bmj.com/site/about/guidelines.xhtml

had a negative influence on outdoors physical activity, as one of the participants illustrated:

I get depressed when I eat less...; The weather is boring and the darkness too and I do not have so many social contacts; When I cook and the kids do not eat that much, I begin eating instead of exercising

It was stated that after some years without exercise, it was difficult to establish a routine and feel motivated to exercise regularly, and this feeling was worse when suffering from pain. One woman explained:

I have lived in Sweden for 8 years, I have four children, and I have not exercised in these years.

A male participant expressed:

Even though I'm on sick-leave, preferred to do other activities at home and do not have time to exercise.

C. Experienced obstacles and facilitators for maintaining lifestyle modifications following the intervention (session six to seven)

C.1. The paradox: raised awareness but still no lifestyle modifications

Despite the raised consciousness concerning the health benefits from changing dietary habits and increasing level of physical activity after the Intervention –especially the women – described that they continued eating sweets and pastries mainly when they were alone, and copiously, large portions when sharing with family and relatives. They found it difficult to lose weight, which they experienced, was related to social/family pressure. Most participants considered the goal of 10000 steps/day to be demanding because of lack of time to exercise

For peer review only

and not feeling comfortable to go for a walk alone.

In fact, some of the women disliked go to the gym, they considered the gym as a "stuffy place";

Further, they stated it was difficult to be alone there.

> *We would like to go to places only for women where we can exercise and do sports. These facilities need to be located near where we live; otherwise, it is not possible for us to go there.*

C.2. Changing lifestyle habits together

In some of the families, spouses were involved and supported the modifications implemented by their partner. In other families, the sessions influenced the whole family, i.e. controlling their children's intake of sugar, and or preparing some dishes in a healthier way:

> *The whole family has changed dietary habits; My husband now eats only hard rye bread now and no white bread anymore; We eat a lot of vegetables, not like before; We bought whole grain rice and started using it instead; My husband has begun to check calories and ingredients in the food he buys, and now he is aware of the food we should not consume and those we should*

Some participants described that they took regular walks together with family members, some started going to the gym:

> *I have started to walk a lot and be more physically active; I have started with water aerobics three times a week and working out twice a week; I also walk; I'm doing gymnastics, training 1 hour each day, 3 times a week and really, I feel*

For peer review only - http://bmjopen.bmj.com/site/about/guidelines.xhtml

better inside and outside; I had tried the treadmill and have trained with the machines in the gym. It is necessary to be strong to be able to lose weight and to get a healthier life

Two of the women involved their family members in domestic tasks, facilitating their possibility of having some extra time for PA:

My daughter and I walk together but my steps are more than hers are. I'm walking fast, I usually walk from 11 o´clock to 17; My son is practicing every day with me in the gym, so he helps me; I have lost 5-6 kilos, I think

During the intervention, some participants lost weight after increasing their everyday physical activity, despite some health problems, which is illustrated here:

Before, I weighed 77 kilos, and now I weigh 70 kilos; My goal was weight loss and although I have pain in my knee, I lost 10 kg; I have walked, and I need to continue walking or exercising to lose weight; I have focused on physical training and if I cannot walk, I exercise at home or go swimming

Discussion

Key findings

This is one of the first qualitative studies investigating self-perceived experiences, barriers and facilitators to LSM in Middle Eastern immigrants to Europe, representing a growing population at high risk for T2D. In this study of first-generation Iraqi immigrants to Sweden, family expectations and structural norms were identified as both strong barriers and facilitators for maintaining healthy lifestyle practices. Culturally adapted lifestyle intervention studies have been conducted previously in Europe; however, these studies are few and address the South Asian population.[12-14, 20] This study is novel in that it addresses the Middle Eastern immigrant

For peer review only - http://bmjopen.bmj.com/site/about/guidelines.xhtml

population.

We have previously shown that this culturally adapted RCT study significantly reduced cardiometabolic risk in the intervention as compared to the control group. Further, this study showed positive impact of the intervention and group sessions not only on reported LSM but also on objectively assessed physical activity as well as metabolism.[15, 21]

Lifestyle challenges in the Middle Eastern immigrant population

The previous population based MEDIM study found a considerable proportion of the Iraqi immigrant population at very high risk for developing T2D within a decade.[9] The Middle Eastern population have other cultural aspects and traditions (such as language, food, religion and sociocultural norms) as compared to the South Asian population. Consequently, this RCT intervention study was initiated as a health initiative to prevent the Middle Eastern population from developing cardiometabolic disease; this study successfully reduced the cardiometabolic risk in these participants by combining different strategies.[15]

Different theoretical models link socioeconomic- and cultural factors with poor health. For instance, in the 'Transitional Model', Schlossberg concludes that sets of factors related to contextual- and individual factors as well as environmental support seem to affect how one copes with transition and adaption to a new lifestyle.[22] Neighborhood deprivation predicts incidence of diabetes in refugees in Sweden,[5] which is in agreement with the WHO, stating that social determinants of health such as socioeconomic conditions, culture and social norms impacts health in chronic diseases morbidity.[3] Hence, LSM are fruitless unless environmental, social- and cultural barriers are identified and addressed.[5, 7] Furthermore, the term 'Social

For peer review only - http://bmjopen.bmj.com/site/about/guidelines.xhtml

capital' has been suggested to be associated with health through several different pathways, including psychosocial mechanisms, norms regarding health related behaviors and access to health care, factors that at the population level are important for the maintenance of health and reduced mortality rates.[23] Middle Eastern immigrants to Sweden in general and Middle Eastern women, in particular, are exposed to all these different levels of social determinants, which is reflected by the fact that >60% of first-generation Iraqi immigrants in Malmö report low social capital (assessed as low social participation, low social anchorage, low instrumental- and low emotional support),[24] as well as high rates of morbidity in non-communicable diseases and poor mental health.[25] The low participation in this study could be a reflection of poor social capital, with low societal trust.

Perceptions, experiences and barriers towards healthier lifestyles

Although the awareness of the impact of healthy lifestyles on health, participants experienced difficulties to modify their lifestyles. The participants perceived that over time it was difficult to establish new dietary habits and become more physically active, which is consistent with studies showing that individuals living in socioeconomically vulnerable areas, challenges in achieving modifications are often hindered by contextual and family influences in achieving lifestyle modifications.[26, 27]

The group sessions addressed knowledge gaps regarding how lifestyle influences the risk of developing T2D. However, the participants perceived as difficult to modify dietary preferences, especially when sharing with family and friends. The perceptions that food boiled in water rather than stir fried or unsweetened drinks do not taste good is a challenge to conquer, especially since eating patterns are commonly guided by family and children's

For peer review only - http://bmjopen.bmj.com/site/about/guidelines.xhtml

expectations which in accordance with prior research.[26, 28-30]

Women commonly described feeling alone in their efforts to change their lifestyles. They felt that family expectations such as taking care of the children and managing the household had a negative influence on their ability to be physically active, which is consistent with what has been reported in other studies.[6-8, 18, 25-27, 29, 31, 32]

In the intervention, 10 000 steps per day was set as a daily goal, but it was perceived by most participants, as being unreachable. Beginning with lower step-goals can enhance adherence to the intervention in sedentary groups.[33] Thus, in this study, a reason for not complying with the intervention could be due to the perception that the goals were too difficult to reach.

Iraqi immigrants in Sweden and Pakistani immigrants in Norway show high prevalence of poor mental health and a strong connection between anxiety and depression with physical inactivity.[25, 34] Iraqi born participants in this study experienced that in order to cope with the dark cold climate and depressed mood they overate and preferred snacks instead of regular meals, which is in line with prior studies conducted among immigrant women at risk for T2D.[26, 27, 29, 31]

Facilitators for maintaining lifestyle modifications

Despite living in Sweden for more than two decades over 70% of Iraqi born immigrants in Malmö do not read or write in Swedish, and prefer traditional calorie dense foods,[35] reflecting low level of integration and acculturation.[24] Family traditions may impose cultural and social norms on each individual, being a barrier for successful LSM. On the other hand, our data indicate that strong family support being a corner stone in the Middle Eastern culture

For peer review only - http://bmjopen.bmj.com/site/about/guidelines.xhtml

represents an important health promoting factor. The Transition Model highlights the importance of receiving support from both, family and environment to enhance adaptation, i.e. to cope with the intervention. Individuals move through the transition process, through a series of phases, i.e. “moving in,” “moving through,” and “moving out”,[22] which may apply to our participants who need to cope, adapt, and make decisions across some cultural- and social situations to succeed with LSM.

The participants who managed to change their lifestyle described a profound support and involvement of their family members in this effort, i.e. motivating each other to maintain healthier habits. These findings correspond with previous research, in which, involving family in target interventions promotes healthier eating behaviors, increased physical activity as well as wellbeing among immigrant populations.[6, 26, 27, 29, 30, 36]

The availability for exercise centers, arenas and other physical environments are important contributors to lifestyles changes.[7, 26, 30, 37] In Malmö, there are several gyms for women and in this study, “Hälsans Hus” i.e. gym facilities only for women located in the nearby neighborhood was one of the physical activity centers women were referred to. Nevertheless, the perceived lack of nearby gender specific gym facilities was a common reason among women to continue being physically inactive. Lack of time was another reason for not performing regular physical activity in this study. A previous study conducted in Sweden showed that Middle Eastern women wanted non- strenuous ‘suitable and pleasant’ exercise, i.e., cycling, swimming, and gymnastics and that it is important that the exercise is ’proper’ i.e. not to exercise in front of the men.[32] Hence, close collaboration with the communities and families is mandatory to fully understand and adjust physical activity for different needs among non-westernized female immigrants, as confirmed by previous research.[7, 29, 32, 33, 37]

For peer review only - http://bmjopen.bmj.com/site/about/guidelines.xhtml

Strengths and limitations

Strengths of the study were the group discussions encouraging respondents to explore individual and shared perspectives and views on the intervention and focus attention on possibilities to improve healthy lifestyles. A native Arabic speaker was present in all sessions, and led back- and forward translations when transcriptions were performed, providing accuracy and credibility of the transcribed and analyzed information. In the last session – session #7, the participants provided feedback and addressed how they had handled obstacles and reached their goals throughout the intervention, thus assuring trustworthiness and confirmability of the information collected in this study. The participants did not know each other prior to the participation. Couples who had participated in the RCT study were randomized to either the intervention or control group,[15] thus, avoiding spread of information between the groups (in this qualitative study, only the intervention group was included). Some participants might have feared to express themselves openly in the group sessions. According to culturally adapted interventions,[7] this issue was addressed by having gender specific groups to assure participants as far as possible could feel free to express their opinions. Although men participated to a lower extent than women, both men and women contributed to all themes as illustrated in Figure 1 where sessions 1 & 2 represented the theme "Motivation to lifestyle change"; sessions 3 to 5 "Perceptions, experiences and barriers towards healthier lifestyles"; sessions 6 & 7 "Experienced obstacles and facilitators for maintaining lifestyle modifications following the intervention". In accordance with the Ethics statement, participants could leave the study without any explanation, thus we do not have data on all reasons for leaving; however, a main reason for not participating was not receiving

For peer review only

For peer review only - http://bmjopen.bmj.com/site/about/guidelines.xhtml

permission from work.

Our findings are limited to middle-aged first-generation Iraqi born immigrants rather than younger second generation participants. Further, ethnicity includes a variety of religious beliefs and family history therefore caution needs to be made when interpreting the data and when transferring our findings to other ethnical minority groups. However, the participants in this study have previously been considered representative for the Iraqi immigrant population living in Sweden.[9] Moreover, the findings of barriers to lifestyle change reported here correspond with previous culturally adapted intervention studies of other non-western immigrant populations as referred to above.

Conclusions

Identification of sociocultural barriers and facilitators for LSM in ethnic minority populations at increased risk of T2D are crucial for both health care professionals and society in order to successfully support populations at risk for T2D, and enable them to successfully reach healthier lifestyle practices. The present study highlighted that facilitators for LSM were connected to profound family support. Tailoring interventions to include entire families, i.e., possibilities for childcare, and the involvement of spouse/other significant family or community member in the intervention could be successful strategies for increasing adherence to interventions/programs among Middle Eastern immigrants.

Barriers and facilitators for lifestyle change in ethnic minority populations are crucial for health care and society to acknowledge in the continued effort to promote healthier LSM in vulnerable populations at risk for non-communicable diseases.

For peer review only - http://bmjopen.bmj.com/site/about/guidelines.xhtml

References

1. Statistics Sweden. Available at http:www.scb.se.
2. Bhugra D, Becker MA. Migration, cultural bereavement and cultural identity. *World Psychiatry*. 2005; 4:18-24.
3. Marmot M, Friel S, Bell R, Houweling TA, Taylor S, Health CoSDo. Closing the gap in a generation: health equity through action on the social determinants of health. *Lancet*. 2008; 372:1661-9.
4. Jervelund SS, Malik S, Ahlmark N, Villadsen SF, Nielsen A, Vitus K. Morbidity, Self-Perceived Health and Mortality Among non-Western Immigrants and Their Descendants in Denmark in a Life Phase Perspective. *J Immigr Minor Health*. 2017; 19:448-76.
5. White JS, Hamad R, Li X, Basu S, Ohlsson H, Sundquist J, et al. Long-term effects of neighbourhood deprivation on diabetes risk: quasi-experimental evidence from a refugee dispersal policy in Sweden. *Lancet Diabetes Endocrinol*. 2016; 4:517-24.
6. Jaber LA, Pinelli NR, Brown MB, Funnell MM, Anderson R, Hammad A, et al. Feasibility of group lifestyle intervention for diabetes prevention in Arab Americans. *Diabetes Res Clin Pract*. 2011; 91:307-15.
7. Lirussi F. The global challenge of type 2 diabetes and the strategies for response in ethnic minority groups. *Diabetes Metab Res Rev*. 2010; 26:421-32.
8. Tiedje K, Wieland ML, Meiers SJ, Mohamed AA, Formea CM, Ridgeway JL, et al. A focus group study of healthy eating knowledge, practices, and barriers among adult and adolescent immigrants and refugees in the United States. *Int J Behav Nutr Phys Act*. 2014; 11:63.
9. Bennet L, Groop L, Lindblad U, Agardh CD, Franks PW. Ethnicity is an independent risk indicator when estimating diabetes risk with FINDRISC scores: a cross sectional study comparing immigrants from the Middle East and native Swedes. Prim Care Diabetes. 2014; 8:231-8.
10. Wändell PE, Carlsson A, Steiner KH. Prevalence of diabetes among immigrants in the Nordic countries. *Curr Diabetes Rev*. 2010; 6:126-33.
11. Rawshani A, Svensson AM, Rosengren A, Zethelius B, Eliasson B, Gudbjörnsdottir S. Impact of ethnicity on progress of glycaemic control in 131,935 newly diagnosed patients with type 2 diabetes: a nationwide observational study from the Swedish National Diabetes Register. *BMJ Open*. 2015; 5:e007599.
12. Telle-Hjellset V, Råberg Kjøllesdal MK, Bjørge B, Holmboe-Ottesen G, Wandel M, Birkeland KI, et al. The InnvaDiab-DE-PLAN study: a randomised controlled trial with a culturally adapted education programme improved the risk profile for type 2 diabetes in Pakistani immigrant women. *Br J Nutr*. 2013; 109:529-38.
13. Bhopal RS, Douglas A, Wallia S, Forbes JF, Lean ME, Gill JM, et al. Effect of a lifestyle intervention on weight change in south Asian individuals in the UK at high risk of type 2 diabetes: a family-cluster randomised controlled trial. *Lancet Diabetes Endocrinol*. 2014; 2:218-27.
14. Admiraal WM, Vlaar EM, Nierkens V, Holleman F, Middelkoop BJ, Stronks K, et al. Intensive lifestyle intervention in general practice to prevent type 2 diabetes among 18 to 60-year-old South Asians: 1-year effects on the weight status and metabolic profile of participants in a randomized controlled trial. *PLoS One*. 2013; 8:e68605.
15. Siddiqui F, Kurbasic A, Lindblad U, Nilsson PM, Bennet L. Effects of a culturally adapted lifestyle intervention on cardio-metabolic outcomes: a randomized controlled trial in Iraqi immigrants to Sweden at high risk for Type 2 diabetes. *Metabolism*. 2017; 66:1-13.
16. Paulweber B, Valensi P, Lindström J, Lalic NM, Greaves CJ, McKee M, et al. A European evidence-based guideline for the prevention of type 2 diabetes. *Horm Metab Res*. 2010; 42 Suppl 1:S3-36.
17. Braun V, Clarke V. What can "thematic analysis" offer health and wellbeing researchers? *Int J Qual Stud Health Well-being*. 2014; 9:26152.
18. Vaismoradi M, Turunen H, Bondas T. Content analysis and thematic analysis: Implications for conducting a qualitative descriptive study. *Nurs Health Sci*. 2013; 15:398-405.
19. Saunders B, Sim J, Kingstone T, Baker S, Waterfield J, Bartlam B, et al. Saturation in qualitative

For peer review only - http://bmjopen.bmj.com/site/about/guidelines.xhtml

research: exploring its conceptualization and operationalization. *Qual Quant*. 2018; 52:1893-907.
20. Andersen E, Burton NW, Anderssen SA. Physical activity levels six months after a randomised controlled physical activity intervention for Pakistani immigrant men living in Norway. *Int J Behav Nutr Phys Act*. 2012; 9:47.
21. Siddiqui F, Koivula RW, Kurbasic A, Lindblad U, Nilsson PM, Bennet L. Physical Activity in a Randomized Culturally Adapted Lifestyle Intervention. *Am J Prev Med*. 2018; 55:187-96.
22. Schlossberg N. A model for analyzing human adaption to transition. *Counseling Psychologist*. 1981; 9:2-18.
23. Kawachi I, Kennedy BP, Lochner K, Prothrow-Stith D. Social capital, income inequality, and mortality. *Am J Public Health*. 1997; 87:1491-8.
24. Bennet L, Lindström M. Self-rated health and social capital in Iraqi immigrants to Sweden: The MEDIM population-based study. *Scand J Public Health*. 2018; 46:194-203.
25. Siddiqui F, Lindblad U, Bennet L. Physical inactivity is strongly associated with anxiety and depression in Iraqi immigrants to Sweden: a cross-sectional study. *BMC Public Health*. 2014; 14:502.
26. Schneider S, Diehl K, Görig T, Schilling L, De Bock F, Hoffmann K, et al. Contextual influences on physical activity and eating habits -options for action on the community level. *BMC Public Health*. 2017; 17:760.
27. Pollard SL, Zachary DA, Wingert K, Booker SS, Surkan PJ. Family and Community Influences on Diabetes-Related Dietary Change in a Low-Income Urban Neighborhood. *Diabetes Educ*. 2014; 40:462-9.
28. Aaboud M, Aad G, Abbott B, Abdallah J, Abdinov O, Abeloos B, et al. Measurement of the [Formula: see text] and production cross sections in multilepton final states using 3.2 fb[Formula: see text] of [Formula: see text] collisions at [Formula: see text] = 13 TeV with the ATLAS detector. *Eur Phys J C Part Fields*. 2017; 77:40.
29. Wieland ML, Weis JA, Palmer T, Goodson M, Loth S, Omer F, et al. Physical activity and nutrition among immigrant and refugee women: a community-based participatory research approach. *Womens Health Issues*. 2012; 22:e225-32.
30. Everson-Hock ES, Johnson M, Jones R, Woods HB, Goyder E, Payne N, et al. Community-based dietary and physical activity interventions in low socioeconomic groups in the UK: a mixed methods systematic review. *Prev Med*. 2013; 56:265-72.
31. Sanchón-Macias MV, Bover-Bover A, Prieto-Salceda D, Paz-Zulueta M, Torres B, Gastaldo D. Determinants of Subjective Social Status and Health Among Latin American Women Immigrants in Spain: A Qualitative Approach. *J Immigr Minor Health*. 2016; 18:436-41.
32. Södergren M, Hylander I, Törnkvist L, Sundquist J, Sundquist K. Arranging appropriate activities immigrant women's ideas of enabling exercise. *Womens Health Issues*. 2008; 18:413-22.
33. Musto A, Jacobs K, Nash M, DelRossi G, Perry A. The effects of an incremental approach to 10,000 steps/day on metabolic syndrome components in sedentary overweight women. *J Phys Act Health*. 2010; 7:737-45.
34. Hjellset VT, Bjørge B, Eriksen HR, Høstmark AT. Risk factors for type 2 diabetes among female Pakistani immigrants: the InvaDiab-DEPLAN study on Pakistani immigrant women living in Oslo, Norway. *J Immigr Minor Health*. 2011; 13:101-10.
35. Siddiqui F, Winther V, Kurbasic A, Sonestedt E, Lundgren KB, Lindeberg S, et al. Changes in dietary intake following a culturally adapted lifestyle intervention among Iraqi immigrants to Sweden at high risk of type 2 diabetes: a randomised trial. *Public Health Nutr*. 2017; 20:2827-38.
36. Patel N, Ferrer HB, Tyrer F, Wray P, Farooqi A, Davies MJ, et al. Barriers and Facilitators to Healthy Lifestyle Changes in Minority Ethnic Populations in the UK: a Narrative Review. *J Racial Ethn Health Disparities*. 2017; 4:1107-19.
37. Cleland CL, Tully MA, Kee F, Cupples ME. The effectiveness of physical activity interventions in socio-economically disadvantaged communities: a systematic review. *Prev Med*. 2012; 54:371-80.

For peer review only - http://bmjopen.bmj.com/site/about/guidelines.xhtml

Table 1. Content of the discussions at each focus group session

Session one (Awareness)	The group discussion addressed how unhealthy lifestyle habits predict risk of diabetes and cardiovascular disease, as well as discussions on nutritional contents of different foods.
Session two (Motivation/Getting started)	It focused on understanding the participant's current dietary and PA habits. The *Action Plan* was introduced as a tool that helped to formulate goals for lifestyle change and to support motivation for implementing lifestyle change, i.e. To write the personal goal and describing how a participant would reach it.
Session three (Taking Action /Maintaining the change)	It was designed to get started by putting dietary change into an Action Plan, by stimulating motivation for dietary change and PA and further by discussing how to achieve the postulated goals. The session also included discussions on common cooking methods for Iraqi food and how to make dishes healthier, i.e. consisting of less fat, sugar and more fibers.
Session four (Cooking session)	In the cooking session, participants were encouraged to bring the recipe of their favorite dish. This recipe was then modified by a professional chef at the cooking session to include healthier dishes including more fibers, less fat as well as less carbohydrates in the diet. The goal of this session was to highlight how cooking method, food preparation and portion size were vital components for effective lifestyle change.
Session five (Relapse – Maintaining the change)	To follow-up the action plan, and to identify barriers to its implementation, as well as experiences regarding social support, and family engagement toward the intervention. Facilitated rewrote action plan.
Session six (Review and feedback)	Participants had documented their everyday PA in a diary and this session included discussions on the final outcomes of lifestyle change, importance of maintaining lifestyle change, motivation to set new goals and discussions on external social support/network to reach those goals. The session also included feedback from the participants.
Session seven (Setting goals and Self-empowerment)	This session highlighted the importance of regularly setting new goals and reviewing these goals. This session also focused on empowering the participants and addressed how they had handled barriers and how they had evolved and reached their goals throughout the process.

For peer review only - http://bmjopen.bmj.com/site/about/guidelines.xhtml

Table 2. The analysis process, of meanings and interpretations of Theme B "Perceptions, experiences and barriers towards healthier lifestyles"

THEME	SUBTHEME	Meanings and interpretations
Perceptions, experiences and further barriers for healthier lifestyles	Challenges with regular meals	• Loneliness at home affects willingness to eat regularly<br>• Eating fast food instead of regular meals<br>• Not perceiving the need for regular mealtimes and unestablished mealtime- schedules<br>• Eating repeatedly snacks and sweets<br>• Sometimes overeating or fasting
	Food preparation, taste preferences and portion size	• Preferences for eating food with "good smell and taste" over dishes prepared in a healthier way (boiled)<br>• Difficulties to introduce to the diet healthier food, healthier cooking, and low-calorie products<br>• Custom of eating large portions
	Family traditions and expectations	• The children decide what to eat in the family hindering a healthy diet<br>• Following family traditions when gathering to eat hindering the willingness to eat moderately<br>• Family resistance to exercise<br>• Challenges changing the role of the mother; limiting the mother´s time and possibilities to exercise
	Other barriers influencing motivation level	• Climate and related factors lessening outdoor physical activity and increasing overeating<br>• Depressed mood-isolation affecting willingness to participate in outdoor physical activities<br>• Difficulty to establish an exercise routine after many years of physical inactivity that worsens with concomitant pain

For peer review only - http://bmjopen.bmj.com/site/about/guidelines.xhtml

Table 3. Group sessions with number of female and male participants throughout the intervention

Session	Women	Men	Total/session
Session One: Awareness	14	9	23
Session Two: Motivation/getting started	15	7	22
Session Three: Taking Action /Maintaining the change	14	4	18
Session Four: Cooking session	9	7	16
Session Five: (Relapse – Maintaining the change)	9	3	12
Session Six: Review and feedback)	9	4	13
Session Seven: Setting goals and Self-empowerment	7	3	10

For peer review only

For peer review only - http://bmjopen.bmj.com/site/about/guidelines.xhtml

Author contributions

POC: Wrote and revised the manuscript, analyzed the data and translated the material coming from the group discussions from Swedish to English.

KB: Participated in conducting the group sessions. Participated in data acquisition, reviewing the translated data transcripts, and notes from Sweden to English for data analyses, and data assessment. Contributed with interpretation of the data. Participated in writing and revising the manuscript.

FS: Participated in the design of the study and in conducting the group sessions. Participated in assessing data and interpretation of the data interpretation. Contributed to writing the manuscript.

LB: Designed and conducted the original study, contributed data acquisition, reviewing the translated data transcripts, and notes from Sweden to English for data analyses, data assessment and interpretation of the data. Contributed to writing and revised the manuscript.

Acknowledgements

We wish to acknowledge the invaluable contribution of the study participants. We are grateful to health coach Muna Mohammed, translator ImanYousif, study Nurses Saranda Muhaxheri, Josefin Goode Khan, and Asma Saleh, administrator Sonja Ruhnke and Ida Hagman. We are also grateful to Hälsans Hus, Rosengård, Abena, Viktväktarna, Arbetarnas Bildningsförbund (ABF) and Oatly. We thank Josephine T. V. Greenbrook for her help with the English language in the initial draft of the manuscript.

Data sharing Statement:

Data can be made available upon request to the corresponding author.

For peer review only - http://bmjopen.bmj.com/site/about/guidelines.xhtml

Figure Legend:

Figure 1: Gender participation rate in relation to total number of the sessions during the intervention.

For peer review only

For peer review only - http://bmjopen.bmj.com/site/about/guidelines.xhtml

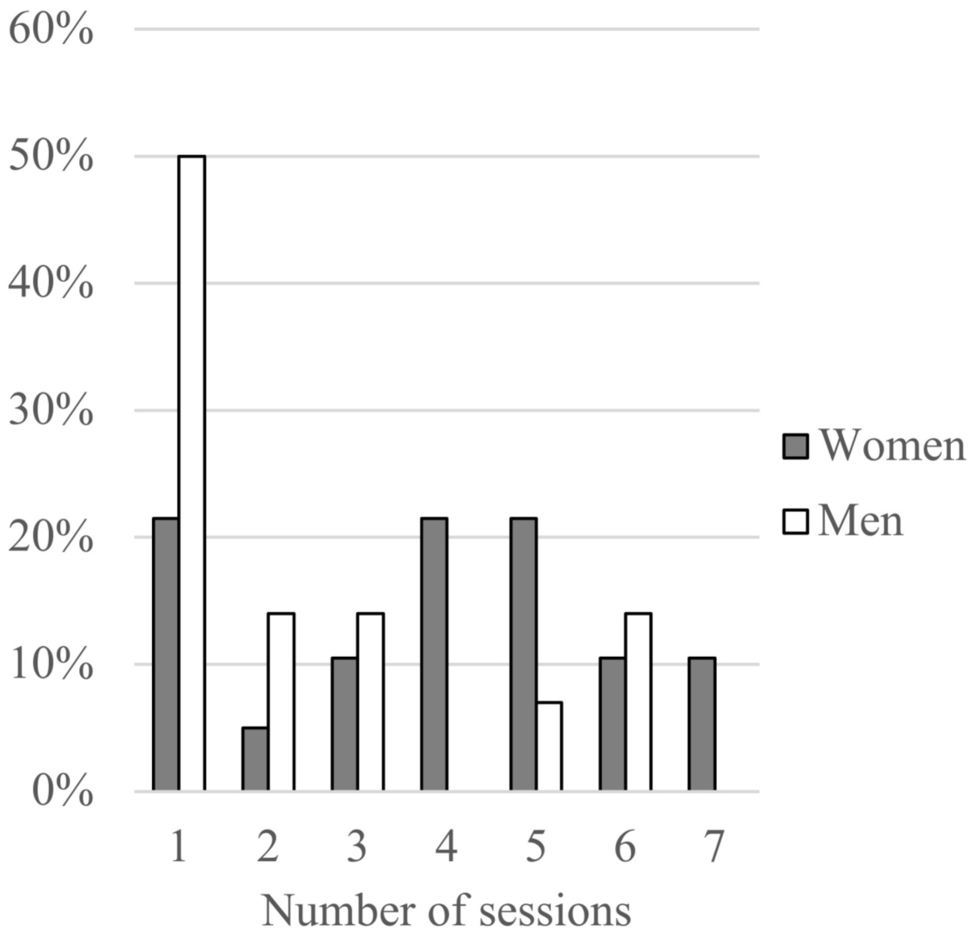

89x89mm (600 x 600 DPI)

Group sessions the MEDIM Intervention study.

The intervention is modified from "The Image toolkit for prevention of type 2 diabetes in Europe: "Take action to prevent diabetes" http://www.image-project.eu/pdf/final_version_of_toolkit-perfect.pdf.

Questionnaires:

- Stage of change [2] or motivation level to enroll in an intervention study, to loose weight and increase their physical activity
- QALY EQ-5D [3]
- Questions about lifestyle habits (diet & exercise, psychological wellbeing)
- Background questions: socioeconomy, current medication and previous diseases, heredity regarding diabetes and cardiovascular diseases, acculturation
- Self-determination theory [4]
- SMART goals that form the basis for the Action Plan Work Sheet (will be developed)
- Physical activity diary
- Food diary

Additional comments:
Smartphone reminders as well as a "Support Line" via telephone.

Group meetings are recorded on tape to document what is said at meetings during discussion.

Session 1. To increase the awareness and knowledge of how lifestyle habits predicts risk of diabetes and cardiovascular disease. Identify health and unhealthy lifestyle habits

A. Identify present lifestyle habits:

1. Let them fill out questionnaires regarding their present food and physical activity (PA) habits. Discuss their answers

1. To increase the awareness and knowledge of how lifestyle habits (food, PA, smoking) predicts risk of diabetes and cardiovascular disease.

a. Inform and discuss healthy level of PA and food intake.
b. Discuss their answers from the questionnaires and ask the participants to identify healthy and unhealthy lifestyle habits regarding food and PA habits.

Session 2. ”To stimulate motivation”:

1. **To stimulate motivation**
    a. Let the participants describe lifestyle habits that they need to change. If they can’t identify them, help them.
2. **Motivation**
    a. Identify and discuss barriers to lifestyle change
    b. Discuss strategies how to handle barriers
    c. Discuss the importance of social support
    d. Discuss pros and cons to lifestyle change (“motivation cross” used in Primary health care)
    e. Identify how important it is to them to change their lifestyle habits and let them fill out the form “Stage of change”
    f. Ask if
        i. They want to continue participating in the intervention
        ii. They want to think about it (participate in the same group again)
        iii. They don’t want to continue (drop)
    g. Give them homework, to take a walk 15 minutes per day.
    h. Provide them with an Action plan work sheet and ask them to set a short term goal, when to do the activity, for how long and how often and what social support they can get. For example take a walk 15 minutes a day when the children are back from school so they can take care of their little 1 year old brother.

Session 3. ”To get started/Taking Action”

Goals:

1. **To create an action plan**
2. **To handle and reduce the risk of relapse**
3. **Homework**

1. **To create an action plan**
    a. Ask the participants to identify lifestyle habits that they want to change
    b. Ask them to fill in the Stage of change form
    c. If they are ready for change ask them what changes they want to do.
    d. Introduce the SMART goal theory and practice to set short and long term goals (physical activity 150 min/week and 5% weight reduction from start). Make them create a ladder of short term goals that will ultimately lead to the big goal. The plan should also include social support they need to achieve and what coping strategies they will use if necessary.

e. Ask participants to write down their own action plan. (This action plan may be revised as they reach their short term goals. A diploma as they reach their short term goals, other ideas of rewards?)

2. To handle and reduce the risk of relapse

a. Discuss the behavioral change wheel, and explain that set backs are a part of the process and an opportunity to learn

b. Discuss and provide tools how to handle and deal with set backs

c. Discuss and provide tools how to handle negative thoughts and stress

d. Identify barriers to change and facilitators for lifestyle change. Identify things that are or are not helping them reaching their goals.

3. Homework

a. Practice action planning, identify negative thoughts and then counter with positive thoughts.

Session four: Cooking session

Session 5 to 7. "Maintaining lifestyle change"

Goals

1. Review the progress of lifestyle change

2. Review the behavioral change process

3. Repeat how to handle relapse

4. Rewrite the action plan and set new goals

1. Review the progress of lifestyle change

a. Go through the homework. What changes have they made? What barriers have they encountered? How did they deal with these barriers? What facilitated to reach goals?

b. Review the SMART goals and their Action plan work sheet. Focus on goals they have achieved. Emphasize the importance of self-monitoring and ask participants to evaluate their progress using their diaries, action plans and smart goals.

2. **Review the behavioral change process and the motivation for behavioral change.** Ask them if it is important to them to implement change and why. Let them fill in a stage of change form.

3. Repeat how to handle relapse.

a. Support successes and discuss setbacks and use them as educational opportunities. Have they made use of coping strategies? If, what has worked? What has not worked? Identify the obstacles that the participants experienced. Discuss in small groups of obstacles that were most relevant to them (emotional, cost-oriented, knowledge-related). In the group, discuss how to get past these barriers. Go through the large group what has been taken up in the small groups. Emphasize the importance of self-monitoring (for example the

use of the accelerometer, etc.). Ask the participants to reflect on what has worked and what has not worked. Make sure the focus is on reaching milestones so that it is easier to build self-confidence and opportunities to achieve success. Ask participants to reflect on their expectations of what to achieve are realistic.

b. Illustrate the importance of periodically review goals and how they evolved. Which goals are they most pleased to have reached? Have there been any unexpected positive changes? Ask participants to identify ways that they can reward themselves.

c. Ask participants to identify positive and negative sources of social support that they had experienced. Did the social support work that they put into their action plan? If not, why? Ask the group to develop more ideas for how social support can act.

4. Rewrite the action plan and set new goals

For peer review only

Reporting checklist for qualitative study.

Based on the SRQR guidelines.

Instructions to authors

Complete this checklist by entering the page numbers from your manuscript where readers will find each of the items listed below.

Your article may not currently address all the items on the checklist. Please modify your text to include the missing information. If you are certain that an item does not apply, please write "n/a" and provide a short explanation.

Upload your completed checklist as an extra file when you submit to a journal.

In your methods section, say that you used the SRQR reporting guidelines, and cite them as:

O'Brien BC, Harris IB, Beckman TJ, Reed DA, Cook DA. Standards for reporting qualitative research: a synthesis of recommendations. Acad Med. 2014;89(9):1245-1251.

		Reporting Item	Page Number
	#1	Concise description of the nature and topic of the study identifying the study as qualitative or indicating the approach (e.g. ethnography, grounded theory) or data collection methods (e.g. interview, focus group) is recommended	3, 7
	#2	Summary of the key elements of the study using the abstract format of the intended publication; typically includes background, purpose, methods, results and conclusions	3, 4
Problem formulation	#3	Description and significance of the problem / phenomenon studied: review of relevant theory and empirical work; problem statement	6, 7
Purpose or research question	#4	Purpose of the study and specific objectives or questions	7
Qualitative approach and research paradigm	#5	Qualitative approach (e.g. ethnography, grounded theory, case study, phenomenolgy, narrative research) and guiding theory if appropriate;	3, 7─10 18─19

For peer review only

		identifying the research paradigm (e.g. postpositivist, constructivist / interpretivist) is also recommended; rationale. The rationale should briefly discuss the justification for choosing that theory, approach, method or technique rather than other options available; the assumptions and limitations implicit in those choices and how those choices influence study conclusions and transferability. As appropriate the rationale for several items might be discussed together.	20—22<br>23
Researcher characteristics and reflexivity	#6	Researchers' characteristics that may influence the research, including personal attributes, qualifications / experience, relationship with participants, assumptions and / or presuppositions; potential or actual interaction between researchers' characteristics and the research questions, approach, methods, results and / or transferability	7—10<br>22
Context	#7	Setting / site and salient contextual factors; rationale	3, 6—8,<br>18—19,21
Sampling strategy	#8	How and why research participants, documents, or events were selected; criteria for deciding when no further sampling was necessary (e.g. sampling saturation); rationale	7—10
Ethical issues pertaining to human subjects	#9	Documentation of approval by an appropriate ethics review board and participant consent, or explanation for lack thereof; other confidentiality and data security issues	7, 10
Data collection methods	#10	Types of data collected; details of data collection procedures including (as appropriate) start and stop dates of data collection and analysis, iterative process, triangulation of sources / methods, and modification of procedures in response to evolving study findings; rationale	3, 7—10
Data collection instruments and technologies	#11	Description of instruments (e.g. interview guides, questionnaires) and devices (e.g. audio recorders)	8-9, 26 Table1

For peer review only

		used for data collection; if / how the instruments(s) changed over the course of the study	Supplementary file
Units of study	#12	Number and relevant characteristics of participants, documents, or events included in the study; level of participation (could be reported in results)	3,5, 7,8, 10-11, 22, 28, figure 1, Table 3
Data processing	#13	Methods for processing data prior to and during analysis, including transcription, data entry, data management and security, verification of data integrity, data coding, and anonymisation / deidentification of excerpts	8—10
Data analysis	#14	Process by which inferences, themes, etc. were identified and developed, including the researchers involved in data analysis; usually references a specific paradigm or approach; rationale	9—10, Table 2
Techniques to enhance trustworthiness	#15	Techniques to enhance trustworthiness and credibility of data analysis (e.g. member checking, audit trail, triangulation); rationale	9—10
Syntheses and interpretation	#16	Main findings (e.g. interpretations, inferences, and themes); might include development of a theory or model, or integration with prior research or theory	10—17 20—21
Links to empirical data	#17	Evidence (e.g. quotes, field notes, text excerpts, photographs) to substantiate analytic findings	10—17, 27
Intergration with prior work, implications, transferability and contribution(s) to the field	#18	Short summary of main findings; explanation of how findings and conclusions connect to, support, elaborate on, or challenge conclusions of earlier scholarship; discussion of scope of application / generalizability; identification of unique contributions(s) to scholarship in a discipline or field	3, 17—19, 20—21 21—23
Limitations	#19	Trustworthiness and limitations of findings	5, 8—10, 19,22—23

For peer review only

Conflicts of interest	#20	Potential sources of influence of perceived influence on study conduct and conclusions; how these were managed	8–9, 21–23
Funding	#21	Sources of funding and other support; role of funders in data collection, interpretation and reporting	2

The SRQR checklist is distributed with permission of Wolters Kluwer © 2014 by the Association of American Medical Colleges. This checklist can be completed online using https://www.goodreports.org/, a tool made by the EQUATOR Network in collaboration with Penelope.ai

For peer review only
